# Oscillatory dynamics of Rac1 activity in *Dictyostelium discoideum* amoebae

**DOI:** 10.1371/journal.pcbi.1012025

**Published:** 2024-12-09

**Authors:** Marko Šoštar, Maja Marinović, Vedrana Filić, Nenad Pavin, Igor Weber

**Affiliations:** 1 Division of Molecular Biology, Ruđer Bošković Institute, Zagreb, Croatia; 2 Department of Physics, Faculty of Science, University of Zagreb, Zagreb, Croatia; Pázmány Péter Catholic University: Pazmany Peter Katolikus Egyetem, HUNGARY

## Abstract

Small GTPases of the Rho family play a central role in the regulation of cell motility by controlling the remodeling of the actin cytoskeleton. In the amoeboid cells of *Dictyostelium discoideum*, the active form of the Rho GTPase Rac1 regulates actin polymerases at the leading edge and actin filament bundling proteins at the posterior cortex of polarized cells. We monitored the spatiotemporal dynamics of Rac1 and its effector DGAP1 in vegetative amoebae using specific fluorescent probes. We observed that plasma membrane domains enriched in active Rac1 not only exhibited stable polarization, but also showed rotations and oscillations, whereas DGAP1 was depleted from these regions. To simulate the observed dynamics of the two proteins, we developed a mass-conserving reaction-diffusion model based on the circulation of Rac1 between the membrane and the cytoplasm coupled with its activation by GEFs, deactivation by GAPs and interaction with DGAP1. Our theoretical model accurately reproduced the experimentally observed dynamic patterns, including the predominant anti-correlation between active Rac1 and DGAP1. Significantly, the model predicted a new colocalization regime of these two proteins in polarized cells, which we confirmed experimentally. In summary, our results improve the understanding of Rac1 dynamics and reveal how the occurrence and transitions between different regimes depend on biochemical reaction rates, protein levels and cell size. This study not only expands our knowledge of the behavior of Rac1 GTPases in *D*. *discoideum* amoebae but also demonstrates how specific modes of interaction between Rac1 and its effector DGAP1 lead to their counterintuitively anti-correlated dynamics.

## Author summary

Oscillations are widespread in biology and occur at the level of ecosystems, physiological processes in organisms, organs and tissues as well as in individual cells. One example is the occurrence of intracellular oscillations of small signaling proteins, the Rho GTPases, which have been identified in a variety of different cell types and species, including yeast, plants and mammals. We show that a protein from this group, Rac1, exhibits polarized and oscillating intracellular patterns in free-living amoeboid cells of the protist *Dictyostelium discoideum*. The active form of Rac1 can either rotate along the cell membrane or move repeatedly from one side of the cell to the other, with a typical period between 2 and 5 minutes. We show by mathematical modeling that biochemical reactions involving proteins that activate and deactivate Rac1 are sufficient to explain the observed dynamics. The modeling shows that the cell size, the amount and the reaction kinetics of the interacting proteins determine whether Rac1 remains homogeneously distributed, polarizes, or oscillates. Since Rac1 is a master regulator of the actin cytoskeleton, our results are important for the quantitative understanding of its role in regulating cell motility.

## Introduction

Cell migration controlled by the cortical actin cytoskeleton is the basis for important biological processes such as embryonic morphogenesis, immune surveillance, wound healing and tumor metastasis. In highly motile cells such as neutrophils, phagocytes and *Dictyostelium discoideum* amoebae, local functional domains within the actin cytoskeleton are rapidly and continuously assembled and disassembled [1,2]. These processes are coordinated spatially and temporally by complex upstream signaling pathways. Understanding these signaling networks requires studying the dynamics *in vivo* of their components, especially the small GTP hydrolases from the Rho family, such as Rac, Rho and Cdc42, which are widely recognized as important regulators of the actin cytoskeleton [3].

As molecular switches, the Rho GTPases cycle between an active GTP-bound state and an inactive GDP-bound state. Activation is facilitated by guanine nucleotide exchange factors (GEFs), and inactivation by GTPase-activating proteins (GAPs). In their active state, Rho GTPases bind and activate effector proteins, including actin-binding proteins, actin polymerases, and their direct regulators. For example, Rac interacts with formins and the Scar/WAVE complex, while Rho regulates myosin II-driven contractility [4–6]. *D*. *discoideum* cells, which share Rho GTPase-dependent signaling mechanisms with mammalian cells, are well suited for the study of these pathways [7,8]. This is particularly true for the three Rac1 isoforms of *D*. *discoideum*, which show more than 80% sequence identity with human Rac1 and have a 100% identical nucleotide-binding domain [7]. In *D*. *discoideum* cells, Rac1 interacts with proteins that play a role in shaping and regulating different structural regions of the actin cortex [7,9]. At the protruding leading edge of polarized motile cells and at the outer edge of circumferential protrusions associated with macroendocytosis, Rac1-GTP activates WASP family proteins and promotes Arp2/3-mediated branched actin polymerization [10]. The non-protruding regions of the cellular cortex are stabilized by proteins that cross-link and bundle actin filaments, such as cortexillins I and II [11]. The bundling ability of cortexillins depends on the formation of a complex with the IQGAP-related protein DGAP1—a process catalyzed by activated Rac1 [12]. Rac1 is therefore of central importance in controlling antagonistic processes that lead to the formation of different actin structures [13,14]. In addition to the WASP and IQGAP protein families, Rac1 has been shown to interact with various other effectors, such as formins, coronins, filamins, PAK kinases and other proteins involved in actin cytoskeleton remodeling [7].

Various imaging techniques have been used to study Rho GTPase activities and their effects on cell polarization, morphology and migration. Most probes for active GTPases are based on fluorescently labeled GTPase binding domains (GBDs) of effector proteins, either alone or incorporated into FRET constructs that detect GTPase activation by RhoGEFs [15–17]. In some experiments, multiple probes were introduced into single cells to observe spatial and temporal correlations between Rac, Rho and Cdc42 activities within protruding lamellipodia [18]. In addition to the “passive” probes that effectively reflect the activity of endogenous GTPases, there are also molecular tools developed for their selective photoactivation, mainly by manipulating associated RhoGEFs [19–22]. In summary, these methods have elucidated the intricate interplay between Rho GTPases and other proteins in actin-driven processes in a range of experimental models and conditions [23,24].

Theoretical modeling has long complemented experimental work to understand the intracellular dynamics of Rho GTPases [25]. In both budding and fission yeast, activated Cdc42 induces stable polarization by promoting actin polymerization and cell outgrowth [26]. Reaction-diffusion models have successfully simulated this polarization by incorporating a positive feedback loop between the activity of Cdc42 and the recruitment of its activating GEF, Cdc24—a mechanism strongly supported by experimental evidence [27,28]. Similar models using mass-conserving and wave-pinning approaches simulated stable polarization of Cdc42 and Rac1 in neutrophils and macrophages [29,30]. Models with GEF-mediated negative feedback have predicted bipolar and oscillatory Cdc42 distributions, in contrast to unipolar patterns in models with only positive feedback [31].

In motile *D*. *discoideum* cells, actin and associated signaling proteins spontaneously form traveling waves at the ventral cell border [32]. Analyzing these patterns, as well as patterns induced by external stimuli, can facilitate the understanding of complex signaling networks and form the basis for the development of advanced theoretical models [33,34]. In this study, we show that active Rac1 and its effector DGAP1 spontaneously form oscillating and rotating patterns in *D*. *discoideum* cell plasma membrane and switch between these dynamic patterns and stationary or irregular distributions over time. To explain these observations, we constructed a reaction-diffusion model centered on the membrane binding and release of Rac1 mediated by the formation of complexes with the cognate GAP and with DGAP1. Our data-driven model faithfully reproduced the experimentally observed dynamics of Rac1 and DGAP1 as well as the correlations between the spatiotemporal patterns of the two proteins. These results suggest that the shuttling of Rac1 between the membrane and the cytoplasm, which is controlled by its interactions with regulatory proteins, adequately explains the observed oscillations.

## Results

### Rac1-GTP shows a repertoire of regular dynamic patterns

**Fig 1:**
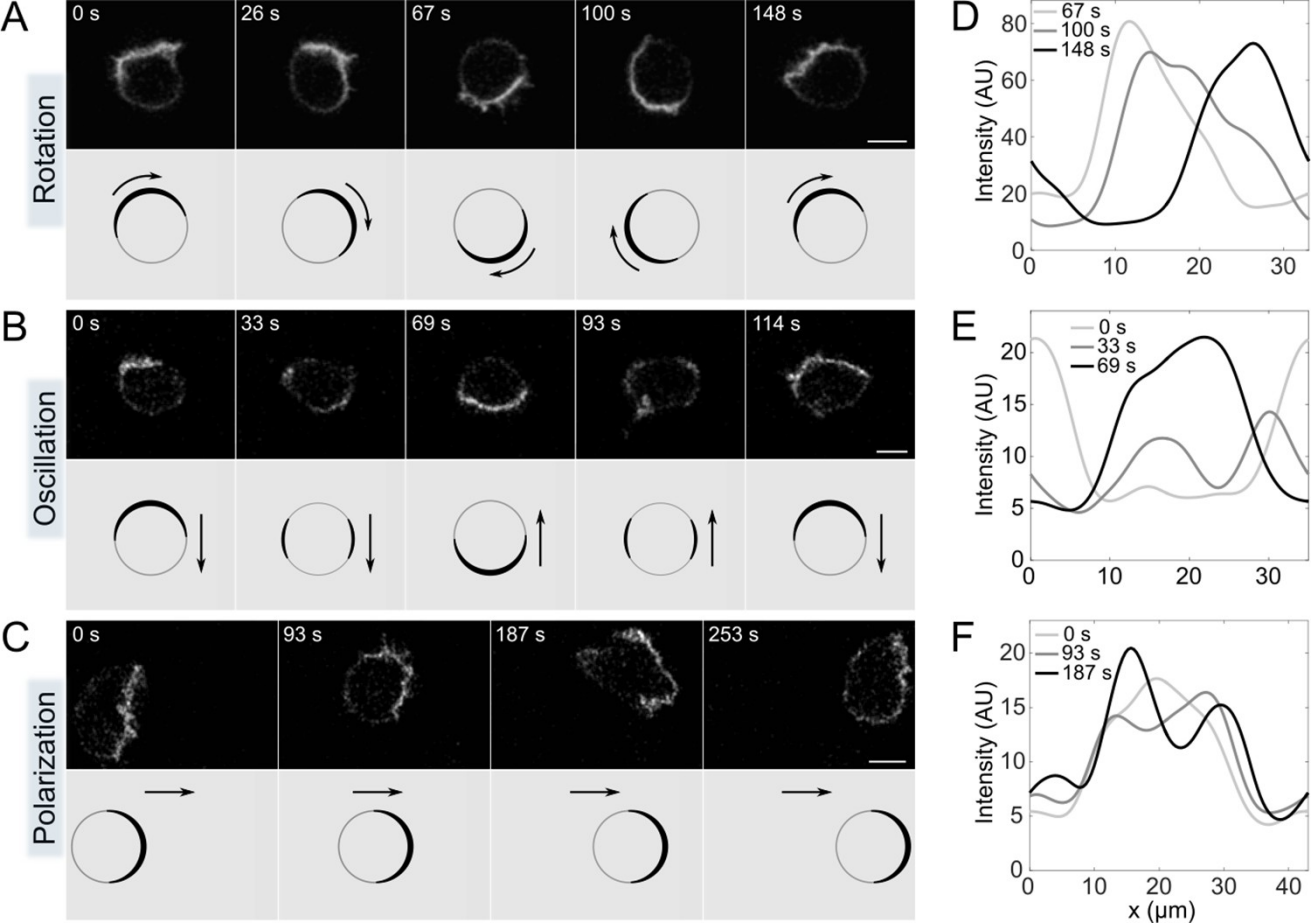
Representative monopolar patterns of Rac1*-enriched cortical domains. (**A**) *Rotating monopole*. Selected frames show the rotation of a Rac1*-enriched membrane domain (top). The corresponding drawing shows the direction of the rotating domain, as indicated by arrows (bottom). (**B**) *Oscillating monopole*. Selected frames show the periodic movement of the Rac1* domain from one side to the opposite side of the cell membrane (top). The corresponding drawing shows the direction of movement of the active domain, marked by arrows (bottom). (**C**) *Stationary monopole*. Selected frames show a Rac1* domain that remains stationary at the front of a stably polarized migrating cell for several minutes (top). The corresponding drawing shows the direction of cell migration indicated by arrows (bottom). (**D-F**) Diagrams showing Rac1* intensity as a function of position on the membrane at three different time points, represented by different shades of gray: (**D**) *Rotating monopole*; (**E**) *Oscillating monopole*; (**F**) *Stationary monopole*. Scale bars: 5 μm.

To characterize the dynamics of Rac1 activity in *D*. *discoideum*, we used confocal microscopy to observe vegetative cells expressing the recently developed fluorescent probe DPAKa(GBD)-DYFP, which specifically binds to Rac1-GTP [35]. For simplicity, we refer to the fluorescent signal corresponding to Rac1-GTP labeled with DPAKa(GBD)-DYFP as Rac1* in the following text. Our observations revealed that Rac1* is mainly localized at the membrane and forms patterns that are either random or ordered and usually persist for several minutes. Of the 85 cells expressing DPAKa(GBD)-DYFP that we observed, 26 showed ordered Rac1* patterns, while the patterns in the remaining 59 cells were classified as random. The ordered patterns can be categorized into three main types: traveling waves (rotations), standing waves (oscillations) and stationary inhomogeneous distributions (polarizations). Each pattern type has either a single Rac1*-enriched membrane domain or two such domains. Based on this criterion, we further classified the observed patterns into six distinct categories: rotating monopoles (n = 14, Fig 1A and S1 Movie), rotating dipoles (n = 3, S1A Fig and S2 Movie), oscillating monopoles (n = 5, Fig 1B and S3 Movie), oscillating dipoles (n = 2, S1B Fig and S4 Movie), stationary monopoles (n = 4, Fig 1C and S5 Movie) and stationary dipoles (n = 1, S1C Fig and S6 Movie). In 21 cells exhibiting rotations and oscillations, we observed 57 distinct periods.

A typical Rac1* traveling wave manifests as a peak distribution that moves along the membrane at a constant speed and has an approximately constant amplitude (Fig 1D). We determined the average cycle duration of these traveling waves to be 190±40 s (mean±std). Conversely, a typical standing wave is characterized by the periodic transfer of Rac1* from one side of the cell to the other. During this transfer, a portion of Rac1* moves along the membrane in two separate sections moving in opposite directions, resulting in an intermediate state characterized by two maxima (Fig 1E, time point 33 s). The average period of the standing waves was 140±20 s. In polarized cells, the Rac1*-enriched membrane domain maintains its position for several minutes, although the intensity profiles in this domain fluctuate on shorter time scales (Fig 1F).

### Rac1-GTP and DGAP1 show similar dynamic patterns that are predominantly anti-correlated

**Fig 2:**
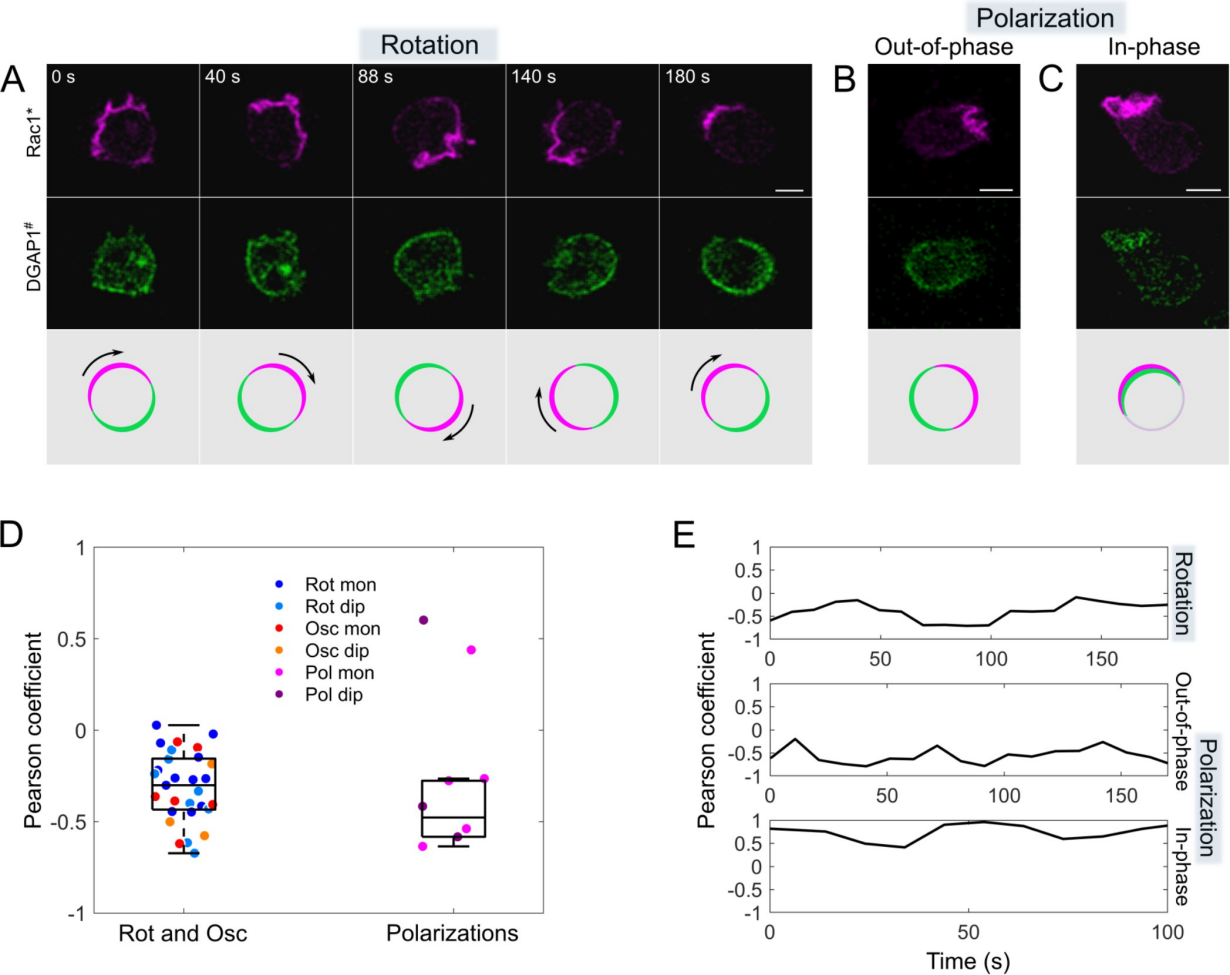
Representative patterns of cortical domains enriched in Rac1* and DGAP1^#^ and their mutual correlation. (A) *Rotating monopole*. An image sequence showing a complete rotation of domains rich in Rac1* and DGAP1^#^. Top: Rac1* signal. Centre: DGAP1^#^ signal. Bottom: a superposition of both fluorescent domains. In areas where Rac1* predominates, the DGAP1^#^ signal is attenuated and vice versa. (B) *Stationary monopole with opposite localization*. Rac1* and DGAP1^#^ are enriched at opposite positions. The image shown is representative of a sequence spanning 170 s. (C) *Stationary monopole with co-localization*. Rac1* and DGAP1^#^ signals are co-localized. The image shown is representative of a sequence extending over 100 s. Scale bars: 5 μm. (D) Quantitative analysis of the correlation between Rac1* and DGAP1^#^ signals. Scatter plots show the Pearson correlation coefficients calculated for the first 120-second interval of 29 cells showing rotating and oscillating patterns (left) and 8 cells showing polarized patterns (right). The overlaid box plots show medians and interquartile ranges for the two cell populations, but the two polarized cells showing positive correlation between Rac1* and DGAP1^#^ signals were excluded from this calculation for polarized cells. (E) Temporal evolution of Pearson correlation coefficients for the three representative cells shown in panels A, B, and C. The coefficients were calculated over sliding 10-seconds intervals throughout the observation period for each cell. The observation periods are different between cells, resulting in unequal time scales of the three plots.

It has been shown that the activated form of Rac1 binds specifically and directly to DGAP1, establishing DGAP1 as a Rac1 effector [12]. To compare the dynamics of Rac1 and DGAP1, we observed cells expressing both DPAKa(GBD)-DYFP (Rac1*) and mRFP-DGAP1, which we refer to as DGAP1^#^ in the remainder of the text. We found that both probes showed rotations (Fig 2A and S7 Movie), oscillations (S2 Fig and S8 Movie) and stable polarizations (Figs 2B, 2C, S9 and S10 Movies). The data show that of the 93 cells observed expressing both probes, 37 cells exhibited ordered patterns, while 56 cells showed random behavior. We observed 20 rotations (12 monopoles and 8 dipoles), 9 oscillations (6 monopoles and 3 dipoles), 6 polarized cells in which the two probes were localized at opposite poles (4 monopoles and 2 dipoles), and 2 cells in which the probes were co-localized (1 monopole and 1 dipole). The average period of the travelling waves was 200±50 s, and that of the standing waves was 160±20 s. In 29 cells co-expressing Rac1* and DGAP1^#^ probes we observed 53 periods of oscillations and rotations. Compared to the dynamics of DPAKa(GBD)-DYFP expressed alone, these results also show that the expression of mRFP-DGAP1 does not significantly affect the dynamics of Rac1*.

In order to analyze the correlation between Rac1* and DGAP1^#^ signals, we calculated the Pearson correlation coefficient (*r*) between the two signals for cells exhibiting ordered behavior (Fig 2D and 2E). For 29 cells showing rotating and oscillating patterns we obtained *r* = −0.30 [−0.43, −0.16] (median [Q1, Q3]), whereas for 6 polarized cells displaying opposite localization of the two probes we obtained *r* = −0.48 [−0.58, −0.28]. Interestingly, the negative correlation between Rac1* and DGAP1^#^ signals was preserved also in 12 cells that displayed random dynamics (*r* = −0.40 [−0.51, −0.32]), suggesting its independency of the large-scale ordered patterns. For the two cells with co-localized signals, we obtained *r* = 0.44 and *r* = 0.60. These rare cases of positive correlation align with our model’s prediction that Rac1* and DGAP1^#^ may co-localize under certain conditions, for example when the concentration of DGAP1 is sufficiently high to saturate its interaction with Rac1. Taken together, our experiments reveal generic, regular patterns in the membrane distribution of active Rac1 in unstimulated vegetative *D*. *discoideum* cells and their close relationship to the corresponding, predominantly anti-correlated patterns of the Rac1 effector DGAP1, which is consistent with our previous results [13,14]. Regular patterns are particularly useful to uncover the processes governing Rac1 dynamics, as they depend on the underlying biochemical reactions. We therefore decided to use these ordered patterns as a basis for investigating the key processes involved in the regulation of Rac1 dynamics through computational modeling.

### Computational model of Rac1 dynamics that takes into account the interaction with its effector DGAP1

To explain the mechanisms underlying the observed spatio-temporal distributions of Rac1* and DGAP1^#^, we propose a theoretical model based on current knowledge of the interactions between Rho GTPases and their regulators and effectors. For practical purposes, we employ a condensed version of the comprehensive model in our numerical simulations. This condensed model was derived iteratively from the full model, aiming to capture the observed dynamics in detail while minimizing the number of reaction terms. We emphasize that the results of the condensed model are equivalent to those of the full model, using a slightly adjusted set of parameter values. A detailed comparison between the two models is provided in Materials and methods (Construction of the theoretical model), and we use the condensed version in the remainder of the Results section.

**Fig 3:**
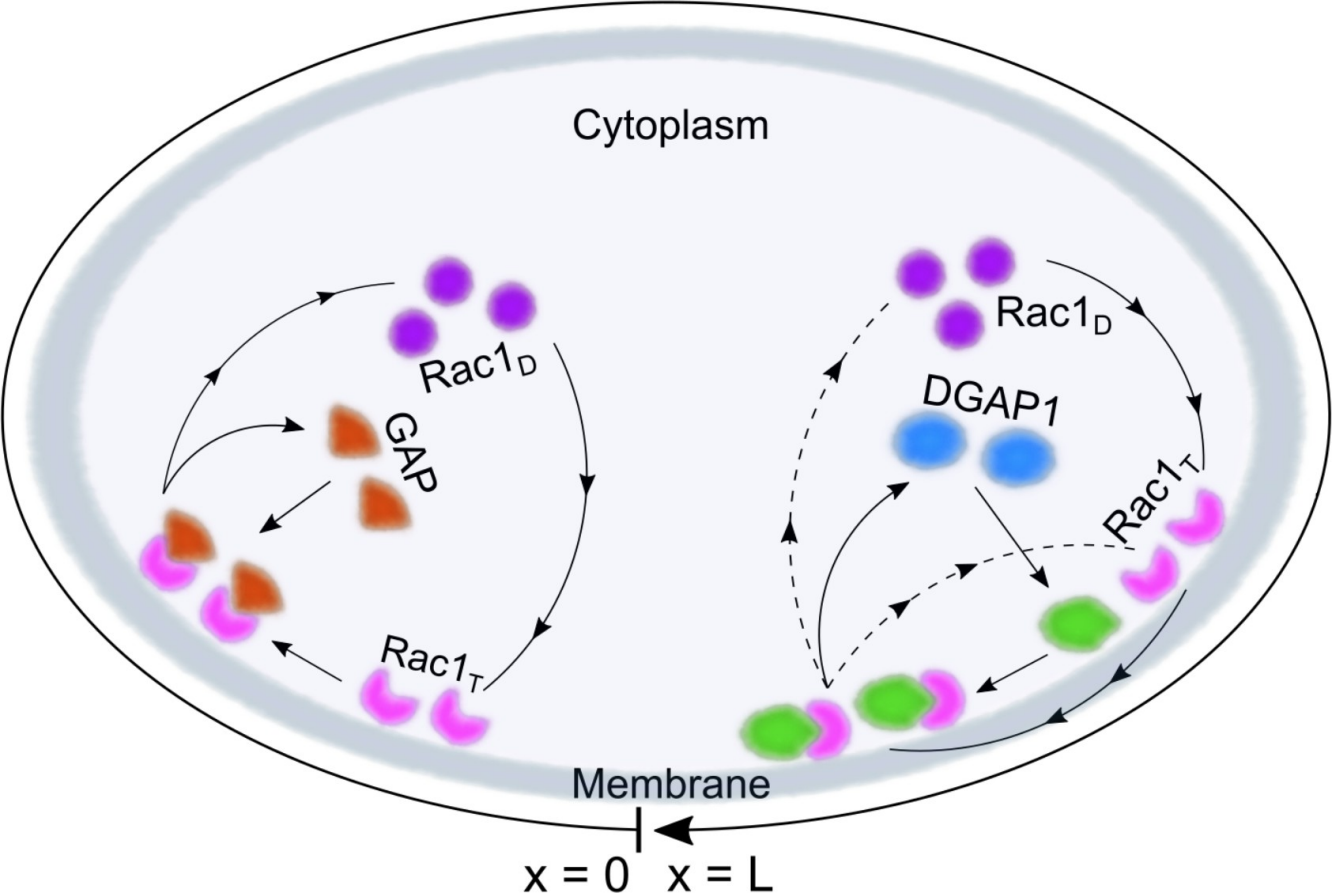
Schematic representation of the protein interaction network. Cycle (i) is shown on the left. Cytoplasmic Rac1_D_ (represented by purple disks) converts to its active form, Rac1_T_, immediately after binding to the membrane. GAP proteins (shown as brown triangles) can bind to active Rac1, leading to its deactivation and the breakdown of the Rac1_T_-GAP complex, resulting in GAP and Rac1 returning to the cytoplasm. Cycle (ii) is shown on the right. DGAP1 proteins from the cytoplasm (blue ovals) bind to the membrane where they form a complex with Rac1_T_. When the Rac1_T_-DGAP1 complex dissolves, DGAP1 moves back into the cytoplasm, while Rac1 is either released into the cytoplasm or remains attached to the membrane.

The reactions involved are graphically represented as two cycles (cycle i and ii) to visually separate the reactions of Rac1-GTP (Rac1_T_ for short) with its regulator GAP from the reactions with its effector DGAP1 (Fig 3). Both cycles in our proposed model involve the binding step of Rac1-GDP (Rac1_D_ for short) to the membrane, coupled with its conversion to Rac1_T_ through the action of a GEF [24,36,37]. In our model, this process also involves autocatalytic GEF-mediated activation of Rac1, which has been experimentally demonstrated for Rho GTPases in various cellular systems and is an established component of most theoretical models of Rho-GTPase dynamics [26,38,39]. In *D*. *discoideum*, RacGEF1 has also been shown to be involved in a positive feedback loop that promotes Rac1 activity [40].

Cycle (i) further describes the binding of cytoplasmic Rac1-specific GAP to membrane-bound Rac1_T_, which promotes its conversion to Rac1_D_, coupled with the release of both proteins into the cytoplasm (Fig 3, left-hand side). Equivalent recruitment of specific GAPs to the cell membrane has also been proposed for active Cdc42 and Ras1 [41,42]. Consistent with this, the localization of *D*. *discoideum* RhoGAP Dd5P4 is largely cytosolic and Dd5P4 is only transiently recruited to the membrane by interacting with activated Rac1 [43,44], in agreement with data available for mammalian RhoGAPs [45,46]. Here we assume that only Rac1_D_ and not Rac1_T_ is extracted from the membrane, so that the Rac1 cycle is unidirectional (there is no branch that brings Rac1_T_ back to the cytoplasm, which would short-circuit the cycle). It has been proposed that tyrosine phosphorylation of membrane-bound Rac1 may facilitate its binding to RhoGDI [47], ensuring the unidirectionality of its GTPase cycle, similar to what has been shown for Ras [48]. Indeed, the release of membrane-bound Rac1_D_ into the cytoplasm has been shown to be favored by an order of magnitude over Rac1_T_ in both GDI-dependent and GDI-independent extraction mechanisms [49,50]. Therefore, it is justified to neglect cytoplasmic Rac1_T_ in the model, even though some minor "leakage" into the cytoplasm might occur.

Cycle (ii) describes the interaction of Rac1_T_ with its effector DGAP1. Our model assumes that DGAP1, in contrast to GAP, first binds to the membrane and then interacts with Rac1_T_ (Fig 3, right-hand side). This assumption is supported by our previous finding that DGAP1 localizes to the cell membrane in cells with actin cytoskeleton disrupted by latrunculin A, in which Rac1 is displaced from the membrane, suggesting a Rac1-independent mechanism for DGAP1 membrane recruitment [14]. We propose two dissociation pathways for the Rac1_T_-DGAP1 complex. The first describes the release of both components into the cytosol in conjunction with the deactivation of Rac1. Several reports have shown that the DGAP1-related protein IQGAP1 promotes the negative regulation of Rac1 activity mediated by RacGAP1 [51]. In addition to the dissociation of the Rac1_T_-DGAP1 complex, which leads to the release of both components into the cytosol, we have introduced another complex dissociation pathway in which Rac1_T_ remains bound to the membrane. Biologically, this corresponds to the possibility that a single Rac1 molecule sequentially binds multiple effectors before being released into the cytoplasm. Other assumptions made during the construction of the condensed theoretical model are described in more detail in the corresponding section of Materials and methods (Construction of the theoretical model).

Based on the described interaction scheme, we have created a mathematical model that includes a series of reactions obeying the law of mass action. The results of computer simulations suggest that pattern formation is based on the accumulation of Rac1 at the membrane and its dissociation from the membrane stimulated by the interaction with GAP or DGAP1. The model reproduces all observed types of Rac1 dynamics: rotations, oscillations and stably polarized states, while preserving the phase relationships between the Rac1 and DGAP1 density profiles. The model is one-dimensional, where the position *x* represents a location at the cell edge and lies within the interval (0, *L*), where the symbol *L* represents the cell perimeter (Fig 3). This is an appropriate choice for cell geometry because active Rac1, which drives cell motility, accumulates predominantly at the cell edge. For simplicity and to facilitate extensive numerical simulations, we have also chosen the one-dimensional geometry for the cytoplasm. However, we also tested a two-dimensional version of the left-hand side of the model that includes the interaction between Rac1 and GAP (cycle i), and obtained qualitatively similar results to those from the 1D version of the model, including traveling and standing wave solutions. The details of this simulation and the obtained results are presented in the Materials and methods section Two-dimensional computational model of Rac1 dynamics.

In our mean-field approach, we describe the distributions of proteins along the cell perimeter by linear densities *ρ*, where the distributions for the membrane-bound active Rac1, DGAP1, Rac1_T_-GAP complex and Rac1_T_-DGAP1 complex are labeled *ρ*_R_,*ρ*_D_,*ρ*_RG_ and *ρ*_RD_, respectively. We also describe the cytoplasmic distributions of inactive Rac1, GAP and DGAP1 by linear densities, denoted by *ρ*_r_,*ρ*_g_ and *ρ*_d_, respectively. Since the positions *x* = 0 and *x* = *L* correspond to the same physical point on the cell membrane, we impose a periodic boundary condition on all densities and their first spatial derivatives, including those at the membrane and in the cytoplasm.

The cycle (i) is described by the following reaction-diffusion equations:

$$\frac{\partial \rho_{r}}{\partial t}=D_{r}\frac{\partial^{2}\rho_{r}}{\partial x^{2}}+k_{3a}\rho_{RG}-\rho_{r}\left(1-\frac{\rho_{R}}{\rho_{Rmax}}\right)(k_{1}+k_{11}\rho_{R}+k_{12}\rho_{RG})+k_{6b}\rho_{RD}$$ (1)

$$\frac{\partial \rho_{R}}{\partial t}=k_{6a}\rho_{RD}-k_{2}\rho_{R}\rho_{g}-k_{5}\rho_{R}\rho_{D}+\rho_{r}\left(1-\frac{\rho_{R}}{\rho_{Rmax}}\right)(k_{1}+k_{11}\rho_{R}+k_{12}\rho_{RG})$$ (2)

$$\frac{\partial \rho_{g}}{\partial t}=D_{g}\frac{\partial^{2}\rho_{g}}{\partial x^{2}}+k_{3a}\rho_{RG}-k_{2}\rho_{R}\rho_{g}$$ (3)

$$\frac{\partial \rho_{RG}}{\partial t}=k_{2}\rho_{R}\rho_{g}-k_{3a}\rho_{RG}.$$ (4)

Here the reactions comprise several steps, with the symbol *k* indicating the reaction rate constants: binding of cytoplasmic Rac1_D_ to the membrane and its activation by GEF, *k*_1_, binding and activation stimulated by membrane-bound Rac1_T_, *k*_11_, and by the Rac1_T_-GAP complex, *k*_12_; binding of cytoplasmic GAP to Rac1_T_, leading to the formation of the Rac1_T_-GAP complex, *k*_2_; deactivation of Rac1 within the Rac1_T_-GAP complex and subsequent release of both proteins into the cytoplasm, *k*_3a_. The model also includes the cytoplasmic diffusion of Rac1 and GAP; the corresponding diffusion constants are denoted by *D*_r_ and *D*_g_, while diffusion along the membrane is neglected. The term (1−*ρ*_R_/*ρ*_Rmax_) in Eqs (1–2) ensures that the membrane density of Rac1 cannot exceed a saturation value, *ρ*_Rmax_.

The cycle (ii) is described by the following reaction-diffusion equations:

$$\frac{\partial \rho_{d}}{\partial t}=D_{d}\frac{\partial^{2}\rho_{d}}{\partial x^{2}}-\rho_{d}\left(1-\frac{\rho_{D}}{\rho_{Dmax}}\right)(k_{4}+k_{41}\rho_{R}+k_{42}\rho_{RG})+(k_{6a}+k_{6b})\rho_{RD}$$ (5)

$$\frac{\partial \rho_{D}}{\partial t}=\rho_{d}\left(1-\frac{\rho_{D}}{\rho_{Dmax}}\right)(k_{4}+k_{41}\rho_{R}+k_{42}\rho_{RG})-k_{5}\rho_{R}\rho_{D}$$ (6)

$$\frac{\partial \rho_{RD}}{\partial t}=k_{5}\rho_{R}\rho_{D}-(k_{6a}+k_{6b})\rho_{RD}.$$ (7)

The reactions of this cycle include: attachment of cytoplasmic DGAP1 to the membrane, *k*_4_, attachment stimulated by Rac1_T_, *k*_41_, and by the Rac1_T_-GAP complex, *k*_42_; formation of the Rac1_T_-DGAP1 complex from membrane-bound DGAP1 and Rac1_T_, *k*_5_; dissociation of the Rac1_T_-DGAP1 complex via two different pathways: release of DGAP1 into the cytoplasm, leaving Rac1_T_ at the membrane, *k*_6a_, or release of both proteins into the cytoplasm, *k*_6b_. We include the diffusion of DGAP1 molecules in the cytoplasm with a diffusion constant *D*_d_, while we neglect their diffusion at the membrane. The term (1−*ρ*_D_/*ρ*_Dmax_) in Eqs (5–6) prevents excessive accumulation of DGAP1 molecules at the membrane with a saturation value of density, *ρ*_Dmax_. Eqs (1)–(7) together represent the joint description of the dynamics of Rac1, GAP and DGAP1.

### Patterns generated by the model

**Fig 4:**
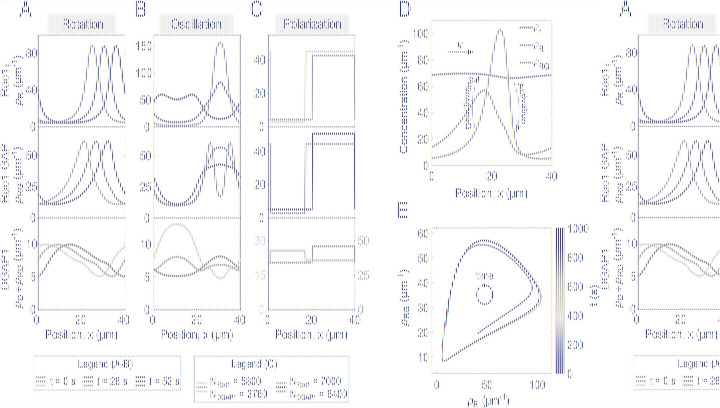
Dynamics of proteins and patterns generated by the model. (**A-C**) Traveling waves, standing waves and polarized patterns of membrane-bound proteins obtained from simulations. For traveling and standing waves, the plots show the molecular density versus position in space for three different time points. For the polarized patterns, the densities are shown for two different total copy numbers of Rac1 and DGAP1 proteins. (**D**) Mechanism of wave propagation of the Rac1 activity. The plot shows linear density profiles of cytoplasmic Rac1_D_ (*ρ*_r_, purple), membrane-bound Rac1_T_ (*ρ*_R_, magenta), and the Rac1_T_-GAP complex (*ρ*_RG_, yellow) in a traveling wave solution of the model. The wave propagates to the right, driven by interplay of three key processes: (1) Recruitment of Rac1_D_ to the membrane and its conversion to Rac1_T_ at the front of the *ρ*_R_ wave, which is driven by the GEF-mediated nonlinear positive feedback and depletes the cytoplasmic Rac1_D_ pool. (2) Deactivation of Rac1_T_ by GAP and replenishment of the cytoplasmic pool of Rac1_D_ at the rear of the *ρ*_R_ wave. (3) The combination of activation at the front and deactivation at the rear establishes a cytoplasmic gradient that drives the forward diffusion of Rac1_D_ in the cytoplasm. (**E**) A phase portrait of Rac1 activity. The plot shows the temporal evolution of linear concentrations of the membrane-bound Rac1_T_ (*ρ*_R_) versus the Rac1_T_-GAP complex (*ρ*_RG_) at a single point in space for the travelling wave pattern. The system trajectory converges towards a stable limit cycle (shown in red). This limit cycle acts as an attractor for all trajectories, regardless of initial conditions. All parameter values used in the simulations are listed in Table 2.

To investigate the behavior of our system, we solved the model numerically for biologically relevant parameters (see Materials and methods section Estimation of the values of parameters used in the simulations). Our results show that after a transient phase, the system converges to either a dynamic (traveling or standing waves) or a stationary regime (Fig 4A–4C). In the case of traveling waves, all membrane-bound protein distributions are characterized by distinct peaks moving at the same constant speed (cf. line profiles for 3 time points in Fig 4A). The profiles of Rac1_T_ and the Rac1_T_-GAP complex form similar patterns, with Rac1_T_-GAP lagging slightly behind. In contrast, Rac1_T_ and DGAP1 are almost anti-correlated, with the minimum of Rac1_T_ near the maximum of DGAP1 and vice versa. Since the traveling waves occur along the closed loop, they correspond to the rotational movement of protein distributions along the cell membrane. For standing wave patterns, Rac1_T_ and DGAP1 are again anti-correlated, while the relationship between Rac1_T_ and Rac1_T_-GAP is more complex than for traveling wave patterns (Fig 4B). Typically, the appearance of a Rac1_T_ density peak is accompanied by a local minimum flanked by two maxima of the Rac1_T_-GAP complex distribution. The subsequent disappearance of the Rac1_T_ peak is accompanied by the formation of a new peak at the position shifted by *L*/2, which corresponds to the opposite side of the cell. Stationary patterns are characterized by sharply separated areas with different protein densities. For Rac1_T_ and the Rac1_T_-GAP complex, there is a large difference in density between the two domains, whereas the difference is smaller for DGAP1 (Fig 4C). Remarkably, the correlation between the distributions of Rac1_T_ and DGAP1 can be either positive or negative, depending on the total number of DGAP1 molecules. In both cases, Rac1_T_ and Rac1_T_-GAP are co-localized.

The formation and propagation of waves are driven by the interplay of three key factors: Rac1 activation at the front, its GAP-mediated deactivation at the rear, and diffusion of Rac1_D_ through the cytoplasm (Fig 4D). Rac1 activation at the *ρ*_R_ wave front is triggered by a positive feedback loop that involves binding of Rac1 to the membrane and the autocatalytic self-recruitment of Rac1 mediated by GEF (*k*_11_ term). The activated Rac1_T_ recruits GAP (*k*_2_ term), leading to the formation of the Rac1-GAP complex. The peak of this complex is located at the back of the Rac1_T_ wave, creating a spatial offset that allows the GAP-mediated deactivation of Rac1 to occur at the rear of the wave, while the front continues to advance through Rac1 activation. The Rac1-GAP complex facilitates the deactivation and release of Rac1 back to the cytoplasm (*k*_3a_ term). The recruitment and activation at the front of the wave deplete the local cytoplasmic Rac1_D_ pool, resulting in the lowest concentration of Rac1_D_ at the front. Conversely, the deactivation and release of Rac1 at the rear of the wave replenish the cytoplasmic Rac1_D_ pool behind the wave. Together, these processes create a cytoplasmic gradient that propels Rac1 forward, driving the wave propagation and determining its speed. To quantitatively assess the influence of model parameters on the wave characteristics, we performed a sensitivity analysis. We found that the rate constant for Rac1 deactivation and release, *k*_3a_, has the largest impact on the wave speed (Table 1). This finding suggests that the GAP-mediated deactivation and release of Rac1 back to the cytoplasm is a critical rate-limiting step in the wave propagation mechanism. The rapid recycling of Rac1 through this process is essential for maintaining the cytoplasmic Rac1 gradient and fueling the wave’s forward movement.

**Table 1:** Local sensitivity analysis of the wave speed.

Parameter	Baseline value	Sensitivity, *S*
*k*_3a_	0.45 s^−1^	1.32
*ρ*_Rmax_	200 μm^−1^	−0.94
*ρ*_Gtotal_	30	0.77
*D*_*g*_	8 μm^2^s^−1^	0.45
*k*_4_	0.24 s^−1^	−0.44
*ρ*_Dtotal_	40	−0.43
*ρ*_Dmax_	40 μm^−1^	−0.38
*k*_2_	0.22 μm/s	−0.35
*k*_6b_	0.43 s^−1^	−0.3
*k*_1_	0.02 s^−1^	0.3
*k*_6a_	0.6 s^−1^	0.22
*k*_12_	6∙10^−3^ μm/s	−0.16
*k*_42_	3.6∙10^−3^ μm/s	0.12
*ρ*_Rtotal_	130	0.11
*k*_5_	0.095 μm/s	−0.10
*k*_11_	4∙10^−3^ μm/s	−0.09
*D*_r_	30 μm^2^s^−1^	0.04
*k*_41_	0∙8∙10^−3^μm/s	0.03
*D*_d_	12 μm^2^s^−1^	0.00

Normalized sensitivity coefficients (*S*) for all model parameters, quantifying their influence on the traveling wave speed obtained from linear stability analysis. Positive *S* values indicate a positive correlation with wave speed, while negative *S* values indicate a negative correlation. Parameters are listed in descending order of their absolute sensitivity coefficients. Baseline values for each parameter, around which variations were made, are also included. For the definition of sensitivity coefficients, see the Materials and methods section Sensitivity analysis.

The travelling waves observed in our simulations represent a self-sustaining pattern that emerges from the system’s dynamics. This pattern can be conceptualized as a stable limit cycle, which serves as an attractor in the phase space of Rac1 activity (Fig 4E). The limit cycle depicts the periodic variations of membrane-bound active Rac1_T_ and the Rac1_T_-GAP complex concentrations at a fixed spatial location as the wave propagates. Irrespective of the starting conditions, the system’s trajectory is drawn towards this limit cycle, highlighting its stability and its role as an attractor that shapes the system’s behavior.

### Stability and bifurcation analyses

**Fig 5:**
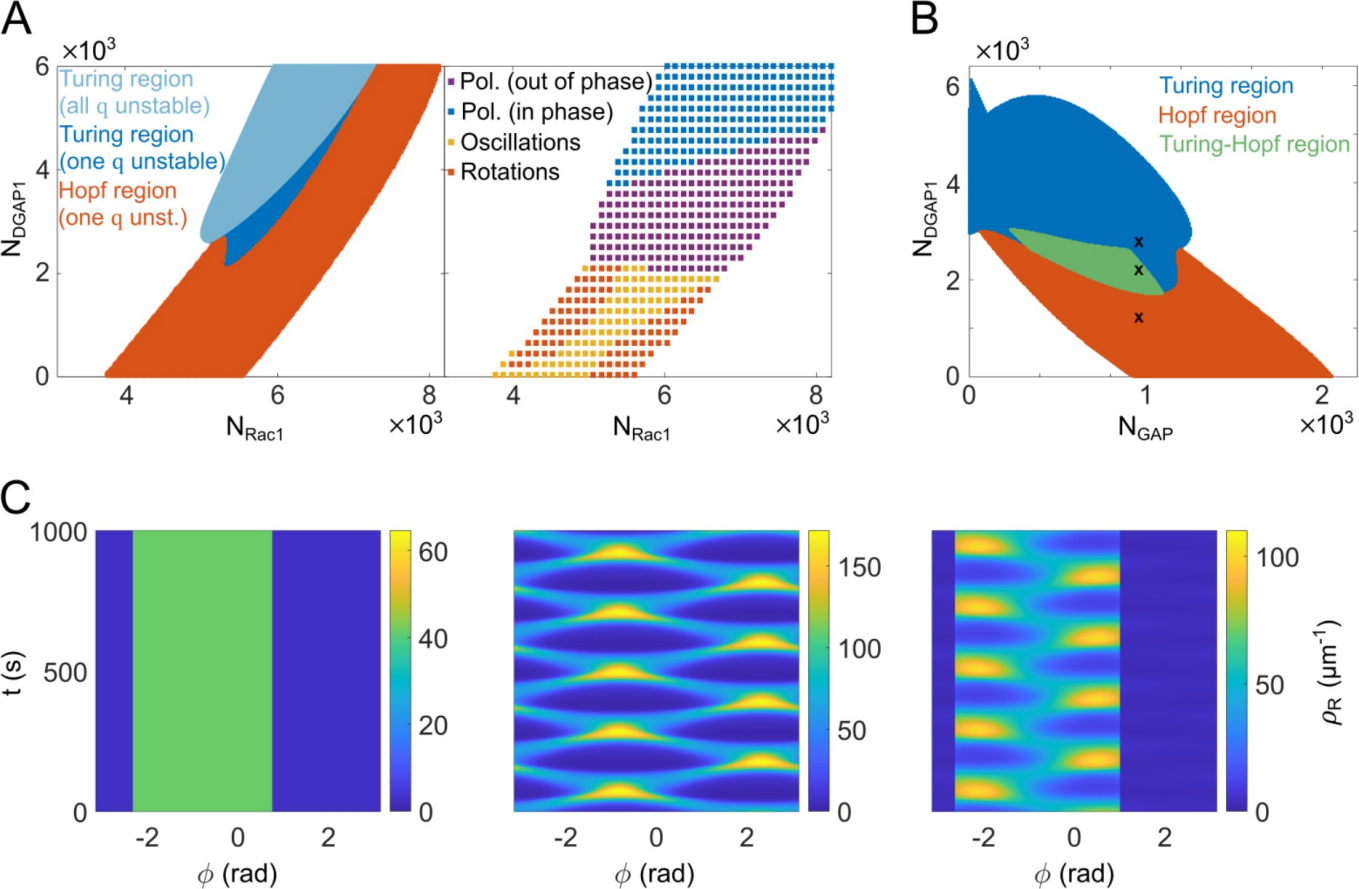
Stability analysis and the pattern formation. (**A**) Stability and dynamic states of the simulated system are presented as functions of the total number of Rac1 and DGAP1 proteins. *Left* Diagram showing different types of instabilities as determined by linear stability analysis. In the uncolored areas of the diagram, small perturbations decay and the system reaches a homogeneous steady state. The blue regions represent the Turing regime, where real positive eigenvalues exist for some wavenumbers *q*, indicating the formation of stationary patterns. The red regions represents the Hopf regime, where complex eigenvalues with positive real parts occur for some wavenumbers *q*, indicating the formation of oscillatory patterns. The intersections of stable, Turing, and Hopf regions mark "co-dimension 2" Turing-Hopf bifurcations. *Right* Diagram of the different dynamic states as a function of the total number of Rac1 and DGAP1 molecules, as determined by examining the final patterns obtained in computer simulations. The uncolored sections of the diagram correspond to the homogeneous states. In both left and right diagrams, the number of GAP molecules is fixed to *N*_GAP_ = 1200. The parameter values used in the simulations are listed in Table 2. (**B**) Diagram showing stability states as a function of the total number of GAP and DGAP1 molecules. The colored regions are defined as in panel A, with an additional Turing-Hopf region (green) where both real and complex eigenvalues with positive real parts coexist for different wavenumbers, suggesting the simultaneous occurrence of stationary and oscillatory patterns. The diagram indicates that GAP is necessary for the occurrence of oscillatory patterns, as evidenced by the absence of Hopf regions along the DGAP1 axis. Conversely, DGAP1 appears to be essential for the occurrence of stationary patterns, shown by the lack of Turing regions along the GAP axis. The number of Rac1 molecules is fixed to *N*_Rac1_ = 5200. (**C**) Representative solutions of the Rac1_T_ dynamics obtained from the model simulations with the total number of GAP molecules fixed at *N*_GAP_ = 960 and with *N*_DAGP1_ corresponding to different stability regions as indicated by the crosses in the panel B. *Left* A stationary Turing pattern obtained with *N*_DGAP1_ = 2800. *Middle* An oscillatory Hopf pattern obtained with *N*_DGAP1_ = 1200. *Right* A superimposition of stationary and oscillatory patterns belonging to the Turing-Hopf region obtained with *N*_DGAP1_ = 2200. The kymographs show the calculated linear Rac1_T_ concentration, *ρ*_R_, along the angular position on the cell perimeter (*ϕ*) versus time (*t*).

To systematically investigate the parameter space, we performed a linear stability analysis of our model. We used Fourier modes to describe the spatial perturbation of the steady state and examined the stability of each mode individually. By varying the total number of Rac1 and DGAP1 molecules (*N*_Rac1_ and *N*_DGAP1_), we discovered two types of instabilities and the corresponding regions in parameter space: Hopf instability (oscillatory) and Turing instability (stationary) (Fig 5A *left*). In the Hopf region, only the first Fourier mode is unstable, indicating that the model can generate oscillatory monopoles under these conditions. Conversely, in the stationary region, we observed areas where either only the first Fourier mode was unstable or all Fourier modes were unstable. This overall instability of all Fourier modes can drive the system into a multipolar state where the number of poles is directly influenced by the initial protein distribution. We then repeated the systematic exploration of the parameter space, but this time using large-scale computer simulations instead of linear stability analysis. The patterns resulting from these simulations were then plotted based on the total number of Rac1 and DGAP1 molecules (Fig 5A *right*). The map derived from the simulation and the map from the linear stability analysis showed significant similarity, except for the high *N*_Rac1_ and high *N*_DGAP1_ region, where the linear stability analysis predicted the formation of periodic patterns but the simulations resulted in stationary patterns.

Linear stability analysis also provides valuable insights into the roles of GAP and DGAP1 in determining the types of patterns that can emerge in our model. By varying the total concentrations of GAP and DGAP1, while keeping the total Rac1 concentration fixed, we have identified four distinct regions in the parameter space (Fig 5B and 5C): the region of stable steady states (no patterns), the region of Turing instabilities (stationary patterns), the region of Hopf instabilities (oscillatory patterns), and the region of Turing-Hopf instabilities (coexistence of stationary and oscillatory patterns). Interestingly, when the total GAP concentration is set to zero, the system only exhibits stable steady states and Turing instabilities, suggesting that GAP is essential for the generation of oscillatory patterns. Conversely, when the total DGAP1 concentration is set to zero, the system only displays stable steady states and Hopf instabilities (and, in some cases, Turing-Hopf instabilities at lower total Rac1 concentrations), indicating that DGAP1 is necessary for the formation of stationary Turing patterns. These findings suggest that the relative abundances of GAP and DGAP1 in the simulated system play a crucial role in determining the type of pattern that emerges. Our theoretical model consists of two distinct interaction cycles: the Rac1-GAP cycle, where cytoplasmic GAP directly binds to the membrane-bound Rac1_T_, and the Rac1-DGAP1 cycle, where DGAP1 first binds to the membrane and then interacts with Rac1_T_. Distinct topologies of these two interaction cycles likely underlie their differential effects on pattern formation. The Rac1-GAP cycle, with its direct negative feedback, may be more conducive to temporal oscillations, whereas the Rac1-DGAP1 cycle, with its membrane-localized interactions, may be more suitable for generating stable spatial patterns.

To explore the impact of the cytoplasmic diffusion of Rac1_D_ and GAP on the pattern formation, we analyzed the stability of steady states for different values of Rac1_D_ and GAP diffusion coefficients by conducting a linear stability analysis on a simplified model focusing on the Rac1-GAP interaction (S3 Fig). Our analysis revealed that Turing patterns emerge even with extremely low Rac1_D_ diffusion coefficients (tested down to *D*_r_ = 10^−6^ μm^2^s^−1^) and zero GAP diffusion. Although this effect is a direct consequence of the postulated immobility of the membrane-bound Rac1_T_ in our model, it demonstrates that even such small difference in diffusion coefficients suffices to break symmetry and enable pattern formation. As GAP diffusion increases (*D*_r_~0.02 μm^2^s^−1^), we observe a transition through a narrow Turing-Hopf region to a more extensive Hopf region (1.5<*D*_g_<20 μm^2^s^−1^), indicating that increased GAP mobility introduces temporal oscillations. At high diffusion coefficients (*D*_g_>20 μm^2^s^−1^), only uniform stable steady states persist. This plateau effect results from a rapid GAP traversal across the domain, leading to Rac1 deactivation and preventing pattern formation.

### Spatio-temporal dynamics of Rac1 activity: a comparison between experimental and modeled results

**Fig 6:**
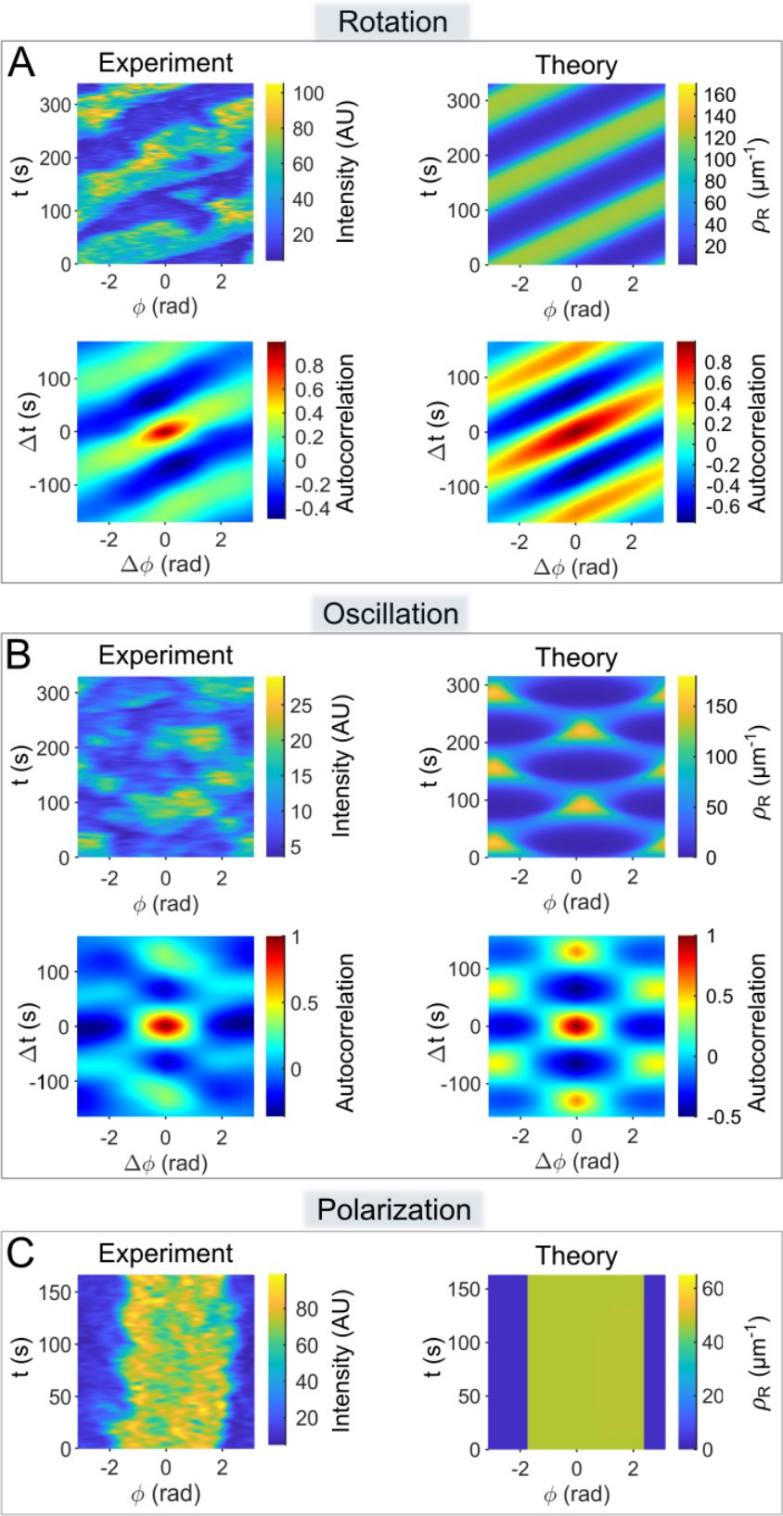
Comparison of the experimentally observed patterns of Rac1* with the patterns of Rac1_T_ concentration (***ρ***_**R**_) derived from computer simulations. In panels A-B, the patterns are shown as kymographs (using the MATLAB colormap: parula) next to the corresponding autocorrelograms (MATLAB colormap: jet). Only kymographs are shown in panel C. The experimental kymographs show the fluorescence intensity measured along the cell perimeter (angular position, *ϕ*) versus time (*t*). Equivalent maps of the calculated linear Rac1_T_ concentration, *ρ*_R_, are shown in the theoretical kymographs. Autocorrelograms represent the self-product of appropriately normalized kymographs that are shifted in both time (Δ*t*) and space (Δ*ϕ*). A precise definition can be found in the Material and methods section. The patterns displayed correspond to the dynamics types shown in Fig 1: (**A**) *Rotating monopole*, (**B**) *Oscillating monopole* and (**C**) *Stationary monopole*. The parameter values used in the simulations are listed in Table 2.

To compare the patterns obtained experimentally and theoretically, we juxtaposed kymographs of the measured Rac1* signal in the membrane and the linear concentration of Rac1_T_ calculated by the model for three typical cases (Fig 6). In addition to kymographs, we also generated corresponding autocorrelograms to facilitate pattern recognition (Figs 6A, 6B and S4A). In the kymographs and autocorrelograms, the traveling waves of the Rac1_T_ distribution are represented as parallel stripes whose slope reflects the wave velocity (Fig 6A). Oscillations lead to regular patterns of patches, while stationary patterns appear as vertical stripes (Fig 6B and 6C). The model reproduces major characteristics of experimentally observed patterns of Rac1 activity. We found that with an appropriate choice of parameter values, the model reproduces the typically measured velocities and wavelengths of traveling waves, the periods of oscillation of monopolar standing waves, and the contrast between and relative widths of active and inactive domains in stationary monopolar patterns. In addition to reproducing the typically observed patterns, we have also successfully simulated less frequently occurring patterns, such as oscillating dipoles (S4A Fig), rotating dipoles (S4B Fig) and stationary dipoles (S4C Fig). In order to make experimentally testable predictions, we investigated how the oscillation patterns depend on the cell perimeter *L*. Linear stability analysis predicts that higher Fourier modes become unstable with increasing *L* (S5A Fig). We tested this prediction by measuring the perimeters of cells that exhibited monopolar and bipolar patterns. Our results showed that monopolar patterns occurred predominantly in small cells, while bipolar patterns occurred in larger cells (S5B Fig).

**Fig 7:**
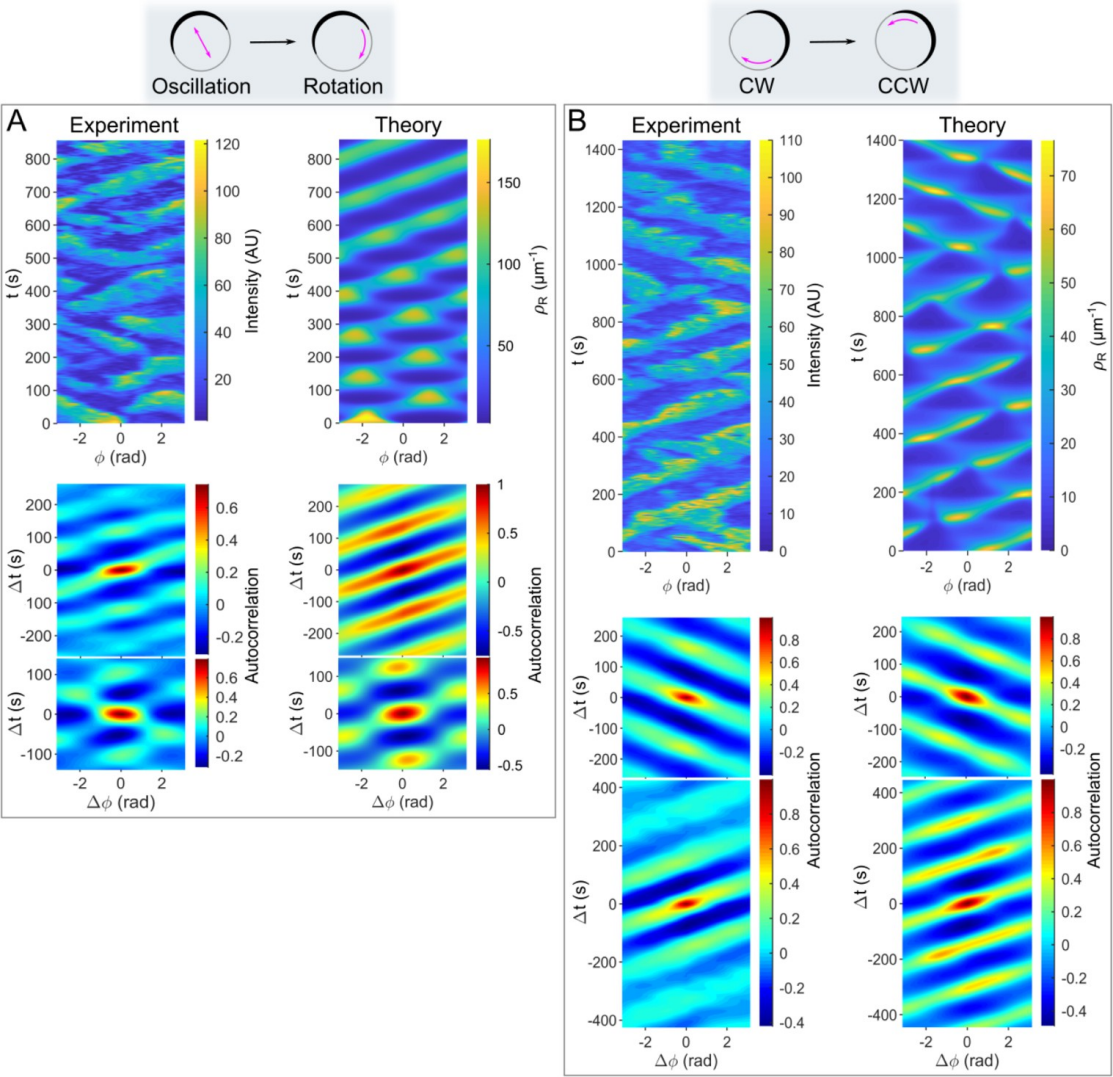
Transitions between different dynamic regimes. (**A**) An experimentally observed transition between the oscillation and the rotation of a single Rac1* domain/monopole (*left*), and the corresponding theoretical simulation of *ρ*_R_ (*right*). (**B**) An experimentally observed transition between the clockwise and counterclockwise rotation of a single Rac1* domain/monopole (*left*) and the corresponding theoretical simulation of *ρ*_R_ (*right*). In this scenario, some parameter values were significantly adjusted from their original estimates to bring the period of the traveling waves in line with the observed experimental data (see Table 2). Kymographs and autocorrelograms are defined as described in Fig 6.

In our experiments, we not only observed transitions between ordered and random patterns, but also transitions between different ordered patterns. For example, we observed a transition from oscillation to rotation, as well as a transition from clockwise to counterclockwise rotation (Fig 7). These results prompted us to investigate whether such transitions can also occur in simulations. Our numerical simulations show that oscillations can transition to rotations and that the duration of these transitions is on the time scale of one period (Fig 7A). In addition, our simulations also show transitions from clockwise to counterclockwise rotations on a similar time scale for different parameter values (Fig 7B). The occurrence of transitions does not strongly depend on the initial conditions, provided they are not in the immediate vicinity of the final stable pattern. We observe transitions even when starting from a slightly perturbed steady state (e.g., with white Gaussian noise and a signal-to-noise ratio of 30 dB). This suggests that the transitions are an intrinsic feature of the system’s dynamics rather than a result of specific initial conditions.

While we do observe transitions from ordered to random patterns of Rac1 activity, the precise role of noise in driving these transitions remains unclear. Several potential sources of disruptive noise could be involved, including stochastic fluctuations due to the low number of molecules, mechanical perturbations caused by changes in cell shape, or extrinsic noise from the cellular environment. Our cross-correlation analysis of Rac1* and DGAP1^#^ signals revealed that the anti-correlation between these signals remains robust even in cells that exhibit random behavior. This finding suggests that the local interactions between Rac1 and DGAP1 are largely independent of the large-scale organization of the cell and points to the dominance of noise at small spatial frequencies in the system. To further explore this result, we performed a power spectrum analysis of the Rac1* signal and observed the dominance of noise power at low spatial frequencies in both ordered and randomly behaving cells (S6 Fig). This result indicates that low-frequency noise is responsible for the disruption of large-scale Rac1* pattern organization, while the small-scale, molecular-level interactions between Rac1 and its regulators remain unaffected.

### Phase relationships between Rac1_T_ and its effector DGAP1

**Fig 8:**
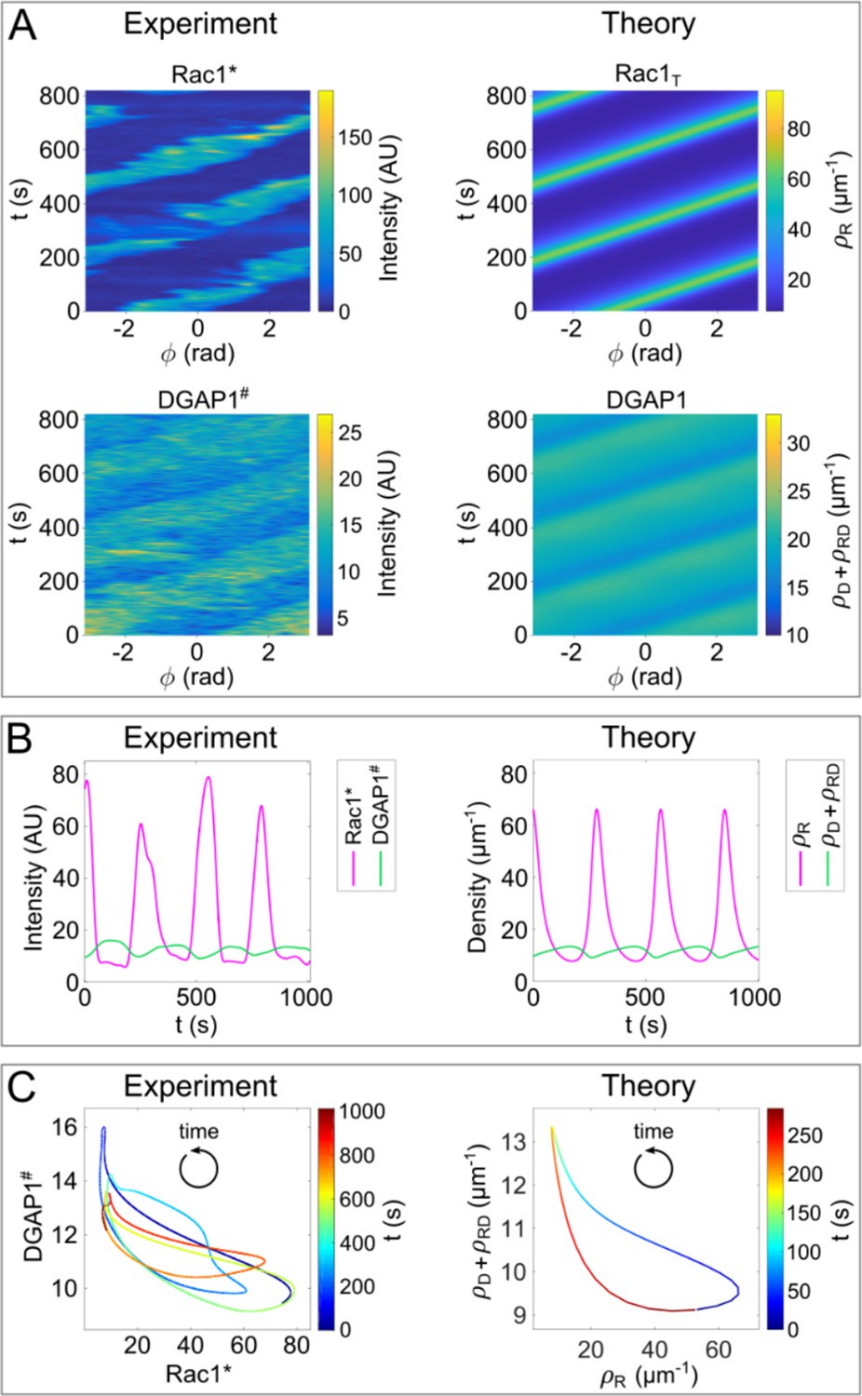
Relationship between the travelling waves of Rac1 and DGAP1. (**A**) Experimentally observed rotational patterns of Rac1* and DGAP1^#^ are displayed next to the corresponding patterns of the linear densities of Rac1_T_ (*ρ*_R_ and DGAP1 (*ρ*_D_+*ρ*_RD_) derived from computer simulations. (**B**) *Left* The average fluorescence intensities of Rac1* and DGAP1^#^, as obtained by principal component analysis of travelling waves, plotted as functions of time. *Right* The time series of *ρ*_R_ and *ρ*_D_+*ρ*_RD_ at a fixed spatial point as derived from numerical simulations. (**C**) Phase portraits created using the data points from (B). The phase portrait derived from the simulated data represents a rotation period (limit cycle). In both experiment and theory, the system moves counterclockwise along a crescent-shaped trajectory. The time course is represented by color coding. The parameters correspond to the values given in Table 2.

Since both DPAKa(GBD)-DYFP and mRFP-DGAP1 interact with active Rac1, it is counterintuitive to observe their fluorescence signals on opposite sides of the cell (Fig 2). To contrast the experimentally observed negative correlation with our theoretical model, we compared kymographs of Rac1* and DGAP1^#^ from microscopy with our theoretical predictions. We emphasize that we compare the experimental distribution of DGAP1^#^, which corresponds to the sum of the free (D) and complexed (RD) forms of DGAP1, to the sum of the model variables *ρ*_D_ and *ρ*_RD_. Both the experimental and theoretical data showed primarily anti-correlated distributions between Rac1 and DGAP1 in oscillating patterns (Fig 8A). To further investigate the relationship between Rac1* and DGAP1^#^ during rotation, we analyzed time series corresponding to specific locations at the cell edge. However, analyzing fluorescence from a single spatial point proved impractical due to excessive noise in the data series. Therefore, we decided to use averaged time series obtained from the data of all spatial points in a single experiment. First, we had to align all time series corresponding to the different locations on the circumference to account for their mutual phase shifts. For this matching, we used principal component analysis (see the corresponding section of Materials and methods). After obtaining the characteristic temporal fluorescence intensity profiles for Rac1* and DGAP1^#^, we compared them to the model simulation (Fig 8B). The calculated profiles agreed remarkably well with the experimental data in terms of periodicity, asymmetry, relative intensities and the phase relationship between the two waves. Using the same experimental data sets, we created a phase portrait showing the counterclockwise evolution along a crescent-shaped trajectory (Fig 8C, *left*). The phase portrait derived from our model simulations mirrors that of the experimental data (Fig 8C, *right*). To capture the correct counterclockwise evolution in the phase portrait, we included the cooperative binding of DGAP1 to the membrane, which is described in the model equations by terms with constants *k*_41_ and *k*_42_. Without these terms, the system always evolved in a clockwise direction, regardless of the choice of other parameter values.

### Dynamics of Rac1-GTP in cells lacking DGAP1

The computed dependence of the emerging Rac1* patterns on the abundance of GAP and DGAP1 (Fig 5B) suggests that, in the absence of DGAP1, the system should only display homogenous stable steady states and oscillations. To test this prediction experimentally, we expressed the Rac1* probe and monitored Rac1 activity in double knock-out mutants lacking both DGAP1 and the closely related protein GAPA. We found that Rac1* also displays wave-like dynamics in these cells (S7 Fig). In 15 DGAP1/GAPA knockout cells expressing the Rac1* probe, we observed 32 periods of oscillations and rotations. This finding is in agreement with the model’s prediction that DGAP1 is dispensable for the occurrence of oscillations and rotations of Rac1* domains. However, we also observed DGAP1/GAPA knockout cells with polarized distributions of Rac1* at the leading edge. This result suggests that other membrane-bound proteins in these cells may take over the role of DGAP1 in the negative regulation of Rac1 activity.

## Discussion

Our study reveals intricate spatiotemporal dynamics of Rac1 activity in *D*. *discoideum*, comprising periodic and irregular patterns as well as transitions between them (Figs 1 and 7). The observed oscillation patterns exhibit periodicities typically between 2 and 5 minutes. Comparable intracellular oscillations of small Rho family GTPases have been observed in a whole spectrum of eukaryotic organisms, from yeast to humans [52]. In budding yeast, clusters of active Cdc42 oscillate with a characteristic period of 4–5 minutes [53]. In fission yeast cells, which grow bipolar, active Cdc42 oscillates between the polarized cell tips with an average period of 5 minutes [54]. In human NK cells, Cdc42 activity oscillates with a periodicity of 5 to 6 minutes [55]. In developing *Xenopus* embryos, active Rho forms waves, while in reconstituted *Xenopus* cortices it organizes in oscillating patches with characteristic periodicities of 1 to 3 minutes [56,57]. Similarly, in plants, the active GTPase Rop1 undergoes oscillatory accumulation and spreading at the tips of growing pollen tubes with a periodicity between 1 and 3 minutes [58]. These consistent periodicities in different organisms suggest that common regulatory mechanisms control the dynamics of Rho GTPases.

Early studies found that the shape changes during locomotion of *D*. *discoideum* cells are not random [59,60]. Quantitative analyses revealed elongations, rotations and oscillations in the shape of both vegetative and starved *D*. *discoideum* cells and showed a correlation with the dynamics of F-actin [61]. The periodicity of the observed rotations and oscillations was between 2.5 and 3.5 minutes, which is consistent with our results. Each type of recurrent pattern lasted 10 to 20 minutes, and the patterns occasionally transitioned spontaneously. However, no bipolar oscillations or rotations of F-actin were detected. Our work and others link Rac activation to actin-driven protrusions in *D*. *discoideum* and other cells [14,22,35,62]. Therefore, it is highly plausible that the dynamic phenomena observed by Maeda and colleagues are related to those described in this study. However, while transitions between different periodic F-actin patterns were hypothesized to occur stochastically, here we show that transitions in active Rac1 patterns can occur within the deterministic framework of our model (Fig 7).

In mutant *D*. *discoideum* cells lacking enzymes that regulate the interconversion of phosphoinositides (PIs), such as PI3-kinase and phosphatase PTEN, the periodic changes in cell shape and the fluid uptake were significantly reduced [61,63]. Subsequently, oscillations in the PI signaling system were observed using fluorescently labeled probe for PIP3 and PTEN in cells immobilized with latrunculin A and treated with caffeine [64]. These studies revealed that the observed oscillations and rotations of membrane domains differentially enriched with PTEN or PIP3 exhibit the properties of a relaxation oscillator [64,65]. Links between PI and Rho GTPase signaling and their oscillations have been suggested in various cellular systems. For example, in neutrophils, PIP3 and active Rac promote actin polymerization, which in turn drives PIP3 and Rac activity through reciprocal positive feedback mechanisms [66–69]. In natural killer cells that form immunological synapses with target cells, the p85a subunit of the PI3-kinase was required to maintain oscillations in Cdc42 activity [55]. Given the striking similarity between the oscillations of PIP3 and Rac1 in *D*. *discoideum*, the question arises whether the established pattern of the former might serve as a template for the spatiotemporal distribution of the latter [70]. Direct experimental testing of this proposition in *D*. *discoideum* is challenging, as the PIP3 waves were observed in immobilized cells treated with latrunculin A and caffeine, a treatment that abolishes the F-actin-dependent cortical localization of Rac1 [14,35]. However, our theoretical studies suggest that the autonomous signaling system, which involves the activation and inactivation of Rac1 and its switching between the membrane and the cytosol, is capable of generating different oscillatory patterns independently of other signaling systems.

Rho GTPases play a crucial role in coordinating the timing, location and nature of supramolecular actin assemblies in motile cells [36,71]. Interestingly, a single Rho GTPase can regulate different cytoskeletal activities by selectively interacting with different downstream effectors [72]. It has been shown that Rac1 in mammals and the related Rac1 in *D*. *discoideum* control the protrusion of lamellipodia and pseudopodia [14,22,35,73], which is facilitated by Rac1-dependent activation of actin polymerases [2,6,7,74]. Besides migration, Rac1 is also involved in the regulation of macroendocytosis, and some of the oscillating domains observed in our experiments clearly represent confocal cross-sections through macropinocytic cups (e.g. S4 Movie) [75,76]. These data indicate that the oscillations might be involved in a periodic sampling of the environment for nutrients and for chemical clues that may trigger chemotaxis [77,78]. However, Rac1 also regulates non-protrusive and contractile areas of the actin cortex, albeit apparently to a lesser extent [13,79–81]. Our study contrasts the dynamics of a Rac1 effector, DGAP1, involved in actin filament entanglement in the posterior and lateral cortex, with the cortical distribution of Rac1*. We confirmed that cortical regions enriched in DGAP1 mostly do not overlap with those enriched in Rac1* [13]. Surprisingly, however, we also observed rare domains enriched in both proteins in polarized cells (Fig 2C), a feature supported by our theoretical model (Fig 5A).

To investigate the observed dynamics from a theoretical perspective, we constructed a reaction-diffusion model based on the generally known interactions between Rac1, its associated regulators from the GAP, GEF and GDI families, and its effector DGAP1. To keep the model manageable, we only explicitly considered the distributions and mutual interactions between Rac1, GAP and DGAP1 (Fig 3), while the activities of the other regulators were indirectly included in the interaction cycle of Rac1. The model contains several nonlinear terms describing feedback mechanisms that facilitate the recruitment of Rac1 and DGAP1 to the membrane. It successfully replicates the spatiotemporal distributions of active Rac1 and DGAP1 in rotational, oscillatory and polar states, transitions between the observed dynamic regimes, and the relative abundances and phase relationships between active Rac1 and DGAP1 on the membrane. Thus, our computational model provides an explanation for the experimentally observed negative correlation between the concentration peaks of active Rac1 and its effector DGAP1 [13,14]. The model predicts that DGAP1 flux is primarily driven by unassisted membrane binding and Rac1-dependent dissociation from the membrane. Consequently, DGAP1 is abundant on the membrane except in regions enriched in Rac1_T_, which binds DGAP1 and releases it into the cytoplasm. The model also predicts a tight coupling between the dynamics of active Rac1 and GAP, a hypothesis that could be explored in future experimental research on RacGAP candidates in *D*. *discoideum*.

Whereas we observed periodic Rac1* patterns in cells devoid of DGAP1, as predicted by the model, the occurrence of polarized Rac1* distributions in these cells suggests that other proteins participate in the negative regulation of Rac1 activity at the membrane of *D*. *discoideum* cells. Since DGAP1 in our model can bind to the plasma membrane independently of Rac1, we expect such auxiliary proteins to share this property. A likely candidate is a recently described RhoGAP CARMIL-GAP, which functions as a GAP for Rac1A in *D*. *discoideum* [43]. Importantly, CARMIL-GAP harbors a pleckstrin homology domain, which can bind phosphatidylinositol lipids within biological membranes, at its N-terminus. While in this report we studied the consequences of DGAP1 removal on the dynamics of Rac1, future investigations could explore analogous effects by altering the abundance of Rac1 itself. Given the presence of three Rac1 isoforms in *D*. *discoideum*, creating single and double Rac1 knock-out mutants could potentially allow for experimental probing of the stability diagram shown in Fig 5A.

In our model, we explicitly considered the interaction of Rac1 with only one of its many effectors [7]. However, the model accounts for positive cooperativity in the binding of Rac1 to the plasma membrane in conjunction with its activation. The underlying mechanism likely involves the interaction of Rac1 with other effectors that promote actin polymerization [40]. It is known that some effectors of Rho GTPases can directly modulate their activity within multi-protein complexes [24]. In general, autocatalytic, GEF-mediated and effector-assisted activation is a universal feature of Rho GTPase regulation in a variety of eukaryotic cells [28,38]. RacGEF1 has also been shown to mediate a positive feedback loop between F-actin and Rac activity in *D*. *discoideum*, although the details of the underlying mechanism are still unclear [40]. Other Rac1-binding proteins such as CIRY can negatively regulate its abundance by acting as Rac1_T_ sequestrators [82]. CIRY proteins, and other Rac1 effectors, probably affect the Rac1 dynamics by influencing the number or free Rac1_T_ molecules locally. Even more importantly, the balance between the number of Rac1_T_ molecules that bind to the Scar/WAVE complex and to the CIRY proteins will influence the dynamics of actin polymerization and feedback loops that depend on F-actin, for instance those that include RacGEF1.

Early models that attempted to explain the symmetry breaking in the distribution of Rho GTPases were inspired by the polarized clustering of Cdc42 in *S*. *cerevisiae* [27,83]. In these seminal studies, it was recognized that autocatalytic GEF-mediated activation is crucial for the polarization of Cdc42 [26]. A little later, active Cdc42 was found to oscillate between transient foci in *S*. *cerevisiae* and between incipient poles in *S*. *pombe* [53,54]. It is often assumed that oscillatory behavior in biological systems requires the presence of a delayed negative feedback loop [84]. Therefore, the oscillations of Cdc42 between the two poles in fission yeast cells were modeled under the assumption that Cdc42 triggers its own elimination through a generic delayed negative feedback mechanism [54]. In budding yeast, negative feedback mechanisms have been proposed to act through Cdc42-mediated activation of a Cdc42-driven GAP or through inhibitory phosphorylation of the Cdc42-specific GEF Cdc24 by the Cdc42 effector Pak1 [53,85–88]. A similar role for Cdc42-specific GEFs and GAPs in the regulation of oscillations by negative feedback was investigated in *S*. *pombe* [41,89]. In our model, Rac1 is negatively regulated through the standard activity of a GAP and a pathway for its detachment from the membrane, mediated by DGAP1.

Our model shares three important features that are essential for the formation of the observed patterns with the conceptual Brusselator model [90]. 1) *Autocatalysis* In both models, autocatalytic steps are crucial for generating oscillations. In our model, a positive feedback loop involving Rac1 recruitment plays a key role in the formation of both stationary and oscillatory patterns. 2) *Substrate depletion* While the Brusselator model, in its original formulation, is not mass-conserving globally due to the constant inflow of substrate and outflow of product, it still relies on the local substrate depletion to generate patterns [90]. Similarly, the local depletion of cytoplasmic Rac1 at the wavefront is crucial for the wave propagation and the formation of spatial patterns. 3) *Diffusion-driven instability* In the Brusselator model, pattern formation arises when the activator diffuses slowly compared to the substrate. Similarly, the membrane-bound active Rac1 (which acts as the activator) is essentially non-diffusible, while the cytoplasmic Rac1 (the substrate) diffuses rapidly, fulfilling the necessary condition for diffusion-driven instability. Moreover, our Rac1 signaling model exhibits a codimension-2 Turing-Hopf bifurcation, similar to what is observed in the Brusselator model. The interplay between Rac1-GAP and Rac1-DGAP1 interaction cycles in our model mirrors the Brusselator’s ability to generate a variety of spatiotemporal patterns, such as polarizations, traveling waves, and standing waves.

It is instructive to compare our theoretical model with the recently published model of Rac and Rho oscillatory dynamics in MDA-MB-231 breast cancer cells [79]. That model includes two GTPases, Rac and Rho, and three other effector/regulator proteins, ROCK, DIA and PAK, that connect them into an interconnected interaction network. Similar to our model, the Bolado-Carrancio model predicts several distinct dynamic regimes, including travelling waves, which depend on the abundances of the effector proteins. However, in contrast to our model, they model the cell as a one-dimensional domain representing the longitudinal axis of the cell and postulate spatially heterogeneous concentration profiles of ROCK and DIA along that axis based on experimental observations. In our model, we do not impose any pre-patterning of GAP or DGAP1 distributions. Instead, the spatial heterogeneity emerges solely from the self-organizing properties of the Rac1-GAP-DGAP1 network.

In summary, our experimental results support the idea that the activities of small Rho GTPases are prone to autonomous, constitutive spatiotemporal oscillations in eukaryotic cells. Our theoretical model for Rac1 dynamics in *D*. *discoideum* cells contributes to the understanding of Rac1 regulation and membrane localization. It supports the idea that regulation of Rho-GTPase signaling activities by GEFs and GAPs can lead to oscillatory dynamics [91]. Future research should investigate the impact of the integrity of the PI-centered signaling network on Rac1 oscillations as well as the contribution of effectors beyond DGAP1 to Rac1 dynamics. Furthermore, our current model does not yet take into account the mechanical forces caused by actin polymerization and membrane deformation [25,92–94]. The functional significance of Rho GTPase oscillations in general remains a controversial topic. Some propose that they represent an exploratory behavior typical of self-organizing biological systems [54,95], while others suggest that the oscillations may be a by-product of the topology of the interaction network [52,96]. In *D*. *discoideum* cells and other related systems, Rac1 oscillations could facilitate the rapid adaptation of cytoskeletal activities to environmental changes [97]. Future studies should investigate the effects of stochasticity on the extent of the different dynamic regimes and the transitions between them. Considering that regulatory and signaling mechanisms involving small Rho GTPases are largely conserved in eukaryotes [7,8,98], we hypothesize that our model is potentially applicable to Rac polarization and oscillations in other systems as well.

## Materials and methods

### Expression vectors and *D*. *discoideum* cell lines

*Dictyostelium discoideum* cells were cultivated in polystyrene culture dishes at 22°C in axenic HL5 medium (Formedium), with the addition of 18 g/l maltose, 50 μg/ml ampicillin and 40 μg/ml streptomycin. We used AX2 cells stably expressing the DPAKa(GBD)-DYFP probe constructed as previously described [14]. DPAKa(GBD)-DYFP probe was also expressed in DGAP1/GAPA double knock-out cells [12]. For the co-expression of DPAKa(GBD)-DYFP and mRFP-DGAP1, AX2 cells stably expressing mRFP-DGAP1 were transfected with the pDEX-DPAKa(GBD)-DYFP vector [14]. Cell transfection by electroporation and clonal selection was performed as described [99].

### Confocal laser scanning fluorescence microscopy

Fluorescence microscopy was performed using a Leica TCS SP8 X microscope equipped with a supercontinuum excitation laser (Leica Microsystems). The excitation wavelengths and detection ranges used for imaging were: (1) 511 nm and 520–565 nm for DYFP, and (2) 575 nm and 585–630 nm for mRFP. The hybrid HyD detectors were operated in gated mode to suppress the parasite reflection from the coverslip surface, and the delay time between excitation and detection was set to 0.3 ns. Only an approximately 1 μm thick section of typically 5–10 μm thick cells was imaged, corresponding to the depth of field of the imaging setup. In total, we observed 85 randomly selected cells expressing DPAKa(GBD)-DYFP and 93 cells co-expressing DPAKa(GBD)-DYFP and mRFP-DGAP1. After selecting a cell that expressed fluorescent probe(s) at moderate levels, it was recorded for at least 10 minutes, and if no obvious patterns were visible, the cell was categorized as having no spatially structured dynamics. Otherwise, the observed pattern was further analyzed. Of the 85 monitored cells expressing DPAKa(GBD)-DYFP, the dynamics in 59 cells were classified as random. Of the 93 monitored cells co-expressing DPAKa(GBD)-DYFP and mRFP-DGAP1, the dynamics were classified as random in 59 cells. In the following analyses of DPAKa(GBD)-DYFP dynamics, the results of both cell types were combined.

### Image processing and correlation analysis

QuimP, a set of plugins for ImageJ (NIH), was used to analyze the intensities of the fluorescent probes on the cell cortex [100]. To remove excess noise from the data, we loaded the output intensities into MATLAB (MathWorks) and processed the data with the smoothing spline function ’csaps’. The resulting variable *I*(*Φ*,*t*) was then color-coded and plotted against the angle *Φ*, which represents the position on the cell membrane, and time *t*. To facilitate the detection of repetitive patterns reflecting protein dynamics, we calculated the autocorrelation function of *I*(*Φ*,*t*). We define the autocorrelation function of fluorescence intensity *I* as

$$A(\Delta \Phi ,\Delta t)=\frac{{\left\langle \delta I(\Phi +\Delta \Phi ,t+\Delta t)∙\delta I(\Phi ,t)\right\rangle }_{\Phi ,t}}{{\left\langle {\delta I}^{2}(\Phi ,t)\right\rangle }_{\Phi ,t}}$$

where $\delta I(\Phi ,t)=I(\Phi ,t)-{\left\langle I(\Phi ,t)\right\rangle }_{\Phi }$, and ⟨ ⟩_Φ,*t*_ denotes the average over the angle and time.

The Pearson correlation coefficient (*r*) between the Rac1* and DGAP1^#^ signals for each cell was computed by applying the MATLAB function ’corr2’ to the first 120 seconds of each recorded time series (Fig 2D). To examine the temporal evolution of the correlation, *r* was computed over sliding 10-seconds intervals throughout the observation period by applying ’corr2’ to successive 10-second portions of the *I*_Rac1*_ and $I_{{DGAP1}^{\#}}$ intensity matrices (Fig 2E).

### Numerical methods

For the numerical calculations, the spatial domain was discretized into 100 uniformly distributed points given by the vector *x* = (*x*_1_,…,*x*_100_), where each component has a value *x*_*i*_ = (*i*−1)∙*h*, with index *i* = 1,…,100 and distance between adjacent spatial points *h* = *L*/100. In this case, the spatial derivatives have an approximate value:

$$\frac{\partial^{2}\rho }{\partial x^{2}}(x_{i},t)=\frac{\rho_{i+1}(t)-2\rho_{i}(t)+\rho_{i-1}(t)}{h^{2}}\;for\;i=2,\ldots ,99$$

$$\frac{\partial^{2}\rho }{\partial x^{2}}(x_{1},t)=\frac{\rho_{2}(t)-2\rho_{1}(t)+\rho_{100}(t)}{h^{2}}$$

$$\frac{\partial^{2}\rho }{\partial x^{2}}(x_{100},t)=\frac{\rho_{1}(t)-2\rho_{100}(t)+\rho_{99}(t)}{h^{2}}.$$

Here, the vector $\rho ={\left(\rho_{r},\rho_{R},\rho_{g},\rho_{RG},\rho_{d},\rho_{D},\rho_{RD}\right)}^{T}$ represents the densities of the protein species. The periodic boundary condition used in this study is included in the equations for the boundaries at *i* = 1 and *i* = 100. Consequently, each partial differential equation was replaced by a system of 100 coupled ordinary differential equations. We solve such a system using MATLAB solver ’ode15s’, a variable-step, variable-order method based on backward difference formulas.

### Linear stability analysis

To investigate the nature of the solutions in larger parts of the parameter space, we performed a linear stability analysis. The original reaction-diffusion system, given by Eqs (1)–(7), can be expressed in a compact form

$$\partial_{t}\rho =D\partial_{x}^{2}\rho +f(\rho ),$$ (M1)

where *f*:ℝ^7^→ℝ^7^ is a nonlinear function given by:

$$f=\left(\begin{array}{c} f_{1}\\ f_{2}\\ f_{3}\\ f_{4}\\ f_{5}\\ f_{6}\\ f_{7} \end{array}\right)=\left(\begin{array}{c} k_{3a}\rho_{RG}-\rho_{r}(1-\rho_{R}/\rho_{Rmax})(k_{1}+k_{11}\rho_{R}+k_{12}\rho_{RG})+k_{6b}\rho_{RD}\\ k_{6a}\rho_{RD}-k_{2}\rho_{R}\rho_{g}-k_{5}\rho_{R}\rho_{D}+\rho_{r}(1-\rho_{R}/\rho_{Rmax})(k_{1}+k_{11}\rho_{R}+k_{12}\rho_{RG})\\ k_{3a}\rho_{RG}-k_{2}\rho_{R}\rho_{g}\\ k_{2}\rho_{R}\rho_{g}-k_{3a}\rho_{RG}\\ -\rho_{d}(1-\rho_{D}/\rho_{Dmax})(k_{4}+k_{41}\rho_{R}+k_{42}\rho_{RG})+(k_{6a}+k_{6b})\rho_{RD}\\ D\rho_{d}(1-\rho_{D}/\rho_{Dmax})(k_{4}+k_{41}\rho_{R}+k_{42}\rho_{RG})-k_{5}\rho_{R}\rho_{D}\\ k_{5}\rho_{R}\rho_{D}-(k_{6a}+k_{6b})\rho_{RD} \end{array}\right),$$

and D is 7×7 diagonal matrix containing diffusion constants,

$$D=\left(\begin{array}{ccccccc} D_{r} & & & & & & \\ & 0 & & & 0 & & \\ & & D_{g} & & & & \\ & & & 0 & & & \\ & & & & D_{d} & & \\ & & 0 & & & 0 & \\ & & & & & & 0 \end{array}\right).$$

We have first determined a fixed point of the system, which is referred to as ρ*. For the spatially homogeneous fixed point, Eq (M1) reduces to *f*(ρ*) = 0. These seven equations are linearly dependent, with four of them being independent:

$$k_{3a}\rho_{RG}^{*}-\rho_{r}^{*}(1-\rho_{R}^{*}/\rho_{Rmax})(k_{1}+k_{11}\rho_{R}^{*}+k_{12}\rho_{RG}^{*})+k_{6b}\rho_{RD}^{*}=0$$

$$k_{3a}\rho_{RG}^{*}-k_{2}\rho_{R}^{*}\rho_{g}^{*}=0$$

$$-\rho_{d}^{*}(1-\rho_{D}^{*}/\rho_{Dmax})(k_{4}+k_{41}\rho_{R}^{*}+k_{42}\rho_{RG}^{*})+(k_{6a}+k_{6b})\rho_{RD}^{*}=0$$

$$k_{5}\rho_{R}^{*}\rho_{D}^{*}-(k_{6a}+k_{6b})\rho_{RD}^{*}=0$$

Furthermore, the total number of molecules of each protein remains constant, regardless of whether these molecules are in a complex or free:

$$\rho_{r}^{*}+\rho_{R}^{*}+\rho_{RG}^{*}+\rho_{RD}^{*}=N_{Rac1}/L$$

$$\rho_{g}^{*}+\rho_{RG}^{*}=N_{GAP}/L$$

$$\rho_{d}^{*}+\rho_{D}^{*}+\rho_{RD}^{*}=N_{DGAP1}/L.$$

Here, the symbols *N*_Rac1_,*N*_GAP_, and *N*_DGAP1_ represent the total number of Rac1, GAP, and DGAP1 molecules, respectively. For a given set of parameter values, these equations have a unique solution, which we have calculated using MATLAB.

To assess the stability of the steady state, we have considered the linearized system

$$\partial_{t}\delta \rho =D\partial_{x}^{2}\delta \rho +J\delta \rho ,$$ (M2)

where *δρ* = *ρ*−ρ* represents the deviation from the steady state, and J denotes the Jacobian matrix of *f*, which is defined as

$$J={\left|\left(\begin{array}{ccc} \frac{\partial f}{\partial \rho_{r}} & \cdots & \frac{\partial f}{\partial \rho_{RD}} \end{array}\right)\right|}_{{\rho =\rho }^{*}}.$$

The linearized Eq (M2) can be solved using the ansatz $\delta \rho_{q}\propto e^{\sigma_{q}t+iqx}$. The stability of the steady state depends on the growth rate *σ*_*q*_, whereby a positive value of the real part indicates an unstable state. The symbol *q* stands for the wavenumber, whose possible values are limited by the boundary conditions. For periodic boundary conditions, the wavenumbers are expressed as *q* = 2*nπ*/*L*, where *n*∈{0,±1,±2,…}. By inserting the ansatz into the linearized system, a connection between the values of *σ*_*q*_ and *q* is established:

$$\sigma_{q}\delta \rho_{q}=(J-q^{2}D)\delta \rho_{q}$$

For a given wavenumber, the values of *σ*_*q*_ are therefore the eigenvalues of the matrix $M_{q}=J-q^{2}D$. If the real part, Re*σ*_*q*_, becomes positive when a control parameter is changed and the imaginary part, Im*σ*_*q*_, is not equal to zero, an oscillatory or Hopf bifurcation occurs. If Im*σ*_*q*_ = 0, however, the instability is described as stationary (Turing).

### Construction of the theoretical model

The condensed theoretical model we use to simulate the joint dynamics of Rac1* and DGAP1^#^ (Eqs 1–7) was derived from an extensive theoretical model described by the following system of equations:

$$\frac{\partial \rho_{r}}{\partial t}=D_{r}\frac{\partial^{2}\rho_{r}}{\partial x^{2}}+k_{3a}\rho_{RG}-\rho_{r}\left(1-\frac{\rho_{R}}{\rho_{Rmax}}\right)(k_{1}+k_{11}\rho_{R}+k_{12}\rho_{RG})+(k_{6b}+k_{6d})\rho_{RD}+k_{-1}\rho_{m}$$ (M3)

$$\frac{\partial \rho_{R}}{\partial t}=(k_{6a}+k_{6c})\rho_{RD}+k_{act}\rho_{m}-k_{2}\rho_{R}\rho_{g}-k_{5}\rho_{R}\rho_{D}$$ (M4)

$$\frac{\partial \rho_{g}}{\partial t}=D_{g}\frac{\partial^{2}\rho_{g}}{\partial x^{2}}+(k_{3a}+k_{3b})\rho_{RG}-k_{2}\rho_{R}\rho_{g}$$ (M5)

$$\frac{\partial \rho_{RG}}{\partial t}=k_{2}\rho_{R}\rho_{g}-(k_{3a}+k_{3b})\rho_{RG}$$ (M6)

$$\frac{\partial \rho_{d}}{\partial t}=D_{d}\frac{\partial^{2}\rho_{d}}{\partial x^{2}}-\rho_{d}\left(1-\frac{\rho_{D}}{\rho_{Dmax}}\right)(k_{4}+k_{41}\rho_{R}+k_{42}\rho_{RG})+(k_{6a}+k_{6b})\rho_{RD}+k_{-4}\rho_{D}$$ (M7)

$$\frac{\partial \rho_{D}}{\partial t}=\rho_{d}\left(1-\frac{\rho_{D}}{\rho_{Dmax}}\right)(k_{4}+k_{41}\rho_{R}+k_{42}\rho_{RG})+(k_{6c}+k_{6d})\rho_{RD}-k_{5}\rho_{R}\rho_{D}-k_{-4}\rho_{D}$$ (M8)

$$\frac{\partial \rho_{RD}}{\partial t}=k_{5}\rho_{R}\rho_{D}-(k_{6a}+k_{6b}+k_{6c}+k_{6d})\rho_{RD}$$ (M9)

$$\frac{\partial \rho_{m}}{\partial t}=\rho_{r}\left(1-\frac{\rho_{R}}{\rho_{Rmax}}\right)(k_{1}+k_{11}\rho_{R}+k_{12}\rho_{RG})+k_{3b}\rho_{RG}-k_{act}\rho_{m}-k_{-1}\rho_{m}$$ (M10)

In comparison to the condensed model described by Eqs (1–7), the extensive model contains an additional species, the membrane-bound inactive Rac1 (represented by the linear density *ρ*_m_), along with several additional reactions. The binding of Rac1_D_ to the membrane and its subsequent activation are treated as distinct kinetic processes. The binding rate is described, as before, by the expression $\rho_{r}(1-\rho_{R}/\rho_{Rmax})(k_{1}+k_{11}\rho_{r}+k_{12}\rho_{RG})$, while the activation rate is described by the linear term *k*_act_*ρ*_m_. Additionally, the reverse process, in which Rac1_D_ is released back to the cytoplasm, is governed by *k*_−1_. A term describing dissociation of DGAP1 from the membrane (*k*_−4_) is also included. Two additional pathways for the dissociation of the Rac1-DGAP1 complex are included: one in which both Rac1 and DGAP1 remain on the membrane, *k*_6c_, and another where Rac1 is released to the cytoplasm while DGAP1 stays on the membrane, *k*_6d_. Finally, an alternative dissociation pathway for the Rac1-GAP complex is included where Rac1 is deactivated but remains membrane-bound, thus generating *ρ*_m_ (*k*_3b_). In order to facilitate the computationally demanding exploration of the parameter space, we introduced several approximations to simplify the extensive model (M3-M10), as detailed below.

*Coupling of the membrane recruitment and activation of Rac1* One of the simplifications is the assumption that Rac1 exists in only two states: active when bound to the membrane and inactive when in the cytoplasm [101]. The latter assumption is supported by evidence suggesting that the release of membrane-bound Rac1_D_ into the cytoplasm is favored over Rac1_T_ by an order of magnitude [49,50,102]. To test the implications of the first assumption—that Rac1 is bound to the membrane exclusively in its active form—we compared the behavior of the extensive model with a modified version in which membrane-bound Rac1_D_ (*ρ*_m_) was removed. Consequently, the terms contributing to the production and removal of *ρ*_m_ are absent in the reduced model. In this reduced model, rather than treating Rac1_D_ membrane binding and activation as separate kinetic processes, these steps were simplified into a single lumped process. Our analysis of the reduced model showed that the system’s behavior remains preserved as long as the timescale of the lumped process approximated the combined timescale of binding and activation (calculated as the sum of the individual reciprocal rates). This condition can be achieved by adjusting binding rate constants (*k*_1_,*k*_11_, and *k*_12_) or the total number of Rac1 molecules. Consequently, this simplification does not lead to qualitative changes in system behavior, as the reduced model still produces the same travelling waves, standing waves, and stationary patterns, while preserving the Rac1-DGAP1 relationship. Since the kinetics of GEF-mediated Rac1 activation in our system are unknown and subject to significant variation and modulation [103], we opted to use in the condensed model a lumped process that captures the essential dynamics of the system while requiring fewer reaction terms, parameters, and molecular species.

*Dissociation of the Rac1-DGAP1 complex* There are four possible pathways for dissociation of the Rac1_T_-DGAP1 complex (RD) in the extensive model: $RD\to R+d(k_{6a}),\;RD\to r+d(k_{6b}),\;RD\to R+D(k_{6c})$, and RD→r+D (*k*_6d_). Numerical simulations showed that including the first two pathways (*k*_6a_ and *k*_6b_), which return DGAP1 to the cytoplasm, was necessary to reproduce the experimentally observed anticorrelation between Rac1* and DGAP1^#^. On the other hand, including only the pathways that retain DGAP1 at the membrane (*k*_6c_ and *k*_6d_), did not yield results consistent with experimental observations. We therefore retained only the first two reactions in the condensed model to streamline time-intensive numerical simulations.

*Dissociation of DGAP1 from the membrane* In the extensive model, the binding of DGAP1 to the membrane (*k*_4_) is followed either by the formation of the Rac1_T_-DGAP1 complex (*k*_5_) or by the direct dissociation of DGAP1 from the membrane (*k*_−4_). By varying the values of *k*_5_ and *k*_−4_, we determined that contribution of direct dissociation to the total DGAP1 flux must be less than 5% to preserve the experimentally observed anti-correlation between Rac1* and DGAP1^#^ signals (cf. Fig 8B). Based on this comparison between experimental and theoretical results, we predict that the vast majority of DGAP1 molecules will interact with Rac1_T_ before being released to the cytoplasm. Consequently, we chose to omit the direct dissociation of DGAP1 from the membrane in the condensed computational model to reduce its complexity.

In addition to the nonlinear term describing the autocatalytic GEF-mediated recruitment of Rac1_D_ to the membrane facilitated by Rac1_T_ anchored in the membrane (*k*_11_), we also included a term which describes the cooperative recruitment of cytoplasmic Rac1_D_ by the membrane-bound Rac1_T_-GAP complex (*k*_12_). We found that the relative magnitudes of *k*_11_ and *k*_12_ regulate the shape of the wave profile and the inclusion of *k*_12_ term was necessary to reproduce the precise shape of Rac1_T_ waves, particularly their width. The nonlinear terms characterized by the rate constants *k*_41_ and *k*_42_ describe the cooperative recruitment of cytoplasmic DGAP1 to the membrane by the membrane-bound Rac1_T_ and the Rac1-GAP complex. These terms were introduced to capture the correct counterclockwise evolution in the phase portrait of Rac1* and DGAP1^#^ (Fig 8C). Without these terms, the system always evolved in a clockwise direction, regardless of the choice of other parameter values. Whereas the Rac1-GAP complex can recruit DGAP1 to the membrane (*k*_42_), there is no corresponding term for the recruitment of GAP by the Rac1-DGAP1 complex. This asymmetry arises because in our model, in contrast to DGAP1, GAP cannot bind to the membrane independently but is recruited only through its interaction with the membrane-bound Rac1_T_. We emphasize that the terms involving constants *k*_12_ and *k*_42_ describe cooperative binding of DGAP1 and Rac1_D_ to the membrane at the sites where the Rac1_T_-GAP complex is present and do not imply formation of tripartite complexes. We acknowledge that the introduction of these cooperative binding terms is not based on established biological mechanisms but was driven by the need to replicate the experimentally observed phase portrait behavior and wave profile shapes. However, the hypothetical recruiting capacity of the Rac1-GAP complex could be mediated by the multi-domain structure of GAPs that can act as scaffolds to promote indirect recruitment of Rac1 and DGAP1 [24,45]. Additionally, three *D*. *discoideum* proteins are known to integrate RhoGEF and RhoGAP domains within the same molecule [9].

To restrict maximal densities of Rac1 and DGAP1 at the membrane, the model uses saturation terms 1−*ρ*_R_/*ρ*_Rmax_ and 1−*ρ*_D_/*ρ*_Dmax_ introduced to capture the effect of steric repulsion and space filling by membrane-bound proteins. Our choice to model Rac1 and DGAP1 with separate density constraints rather than summing membrane concentrations of all species stems from significant differences in their molecular sizes (approximately 21 kDa for Rac1 and 95 kDa for DGAP1) and the distinct crowding effects they are likely to experience. This approach is supported by recent findings [104], which demonstrate that the crowding effects on membrane proteins can vary substantially depending on the physical properties of proteins, such as size, shape and charge. Moreover, during the model development process, we explored an alternative approach in which the concentrations of both proteins were summed to represent the total protein density on the membrane. However, we found that this formulation was unable to reproduce certain aspects of the observed Rac1 and DGAP1 dynamics, particularly their mutual correlation. The constants *ρ*_Rmax_ and *ρ*_Dmax_ were not derived from experimental measurements but were instead treated as free model parameters.

### Estimation of the values of parameters used in the simulations

We estimated the average membrane length of the observed cells to be about 40 μm. Small GTPases typically have a molecular mass that varies between 20 and 25 kDa [105], the mass of DGAP1 is 95 kDa [106], and the mass of a RhoGAP protein from *D*. *discoideum*, DRG, is 150 kDa [107]. Using the diffusion coefficient of GFP (27 kDa) measured in the cytoplasm of *D*. *discoideum* cells (27 μm^2^s^-1^) as a reference [108], the value of the Rac1_D_ diffusion coefficient was set to *D*_r_ = 30 *μ*m^2^s^−1^. The Einstein-Stokes equation predicts that the diffusion coefficient of a globular protein should be inversely proportional to its molecular mass (*D* ~ M^−1/3^). However, due to the effects of crowding and the irregular shape of non-spherical proteins, smaller proteins have been found to follow the relationship *D*~*M*^−2^, while the diffusion coefficient for larger proteins (*M*>100 kDa) can become almost independent of *M*[109]. Therefore, we set the values of DGAP1 and GAP diffusion coefficients to *D*_d_ = 12 *μ*m^2^s^−1^ and *D*_g_ = 8 *μ*m^2^s^−1^.

To estimate the total number of molecules for each protein in our one-dimensional system, we need to convert the available information about 3D concentrations into 1D concentrations. Since the molecules usually have greater freedom of movement in the horizontal xy-plane because the cells often lie flat on a surface, the observed protein dynamics tend to be largely independent of the *z*-position within the cell. For this reason, we chose a 100 nm thick horizontal layer to represent the entire cell. Although the choice of layer thickness is somewhat arbitrary, it directly influences the total amounts of protein used in the simulations. Given a concentration of cellular Rac1 of 1 *μ*M [110] and a typical cell radius of 6 *μ*m, we estimate the total number of Rac1 molecules in a slice to be approximately 7000. We did not adjust this value significantly for our simulations, although the spatial domain in our simulations is ring-shaped rather than disk-shaped. We scaled the total number of DGAP1 and GAP proteins according to their size, as we assume that the abundance of a particular protein type is inversely proportional to its size [111].

The dissociation rate constants of the Rac1-containing complexes, *k*_3a_,*k*_6a_ and *k*_6b_, were initially set to 0.5 *s*^−1^, as described in the literature [89,112]. These values were only slightly adjusted in our simulations. To avoid unrealistic concentrations of membrane-bound proteins, we set biologically plausible upper limits for the densities of membrane binding sites for Rac1_T_ (*ρ*_Rmax_) and DGAP1 (*ρ*_Dmax_). Next, we set the membrane binding constant for Rac1, *k*_1_, to match the flux of Rac1 from the cytoplasm to the membrane to values commonly cited in the literature [50]. Similarly, we fitted the nonlinear rate constants *k*_11_ and *k*_12_ considering the steady-state concentrations of Rac1_T_ (of the order of 10 μm^−1^) and Rac1_D_ (of the order of 10^2^ μm^−1^). Similarly, we estimated the initial values for the rate constants that determine the membrane binding of DGAP1, *k*_4_,*k*_41_ and *k*_42_.

We then determined the rate constants for the binding reactions of Rac1_T_ to GAP, *k*_2_, and to DGAP1, *k*_5_, to obtain rates of the order of 1 *s*^−1^ at steady state [113]. Based on these preliminary estimates, we adjusted all reaction constants to successfully replicate the measured dynamical properties of the system, such as wave profiles, periods, and the phase relationship between the Rac1_T_ and DGAP1 signals. The numerical values for all parameters used in the simulations are listed in Table 2.

**Table 2:** Numerical values of parameters used in numerical simulations.

Parameter	Initial values	Values used in individual figures (without physical units)
*ρ*_Rmax_	200 μm^−1^	−
*ρ*_Dmax_	40 μm^−1^	(7B) 70
*D*_r_	30 μm^2^s^−1^	(7B) 70
*D*_g_	8 μm^2^s^−1^	(7B) 40
*D*_d_	12 μm^2^s^−1^	(7B) 30
*k*_1_	2∙10^−2^s^−1^	(S4B) 4.8∙10^−2^; (7A) 3∙10^−2^; (7B) 1.57∙10^−2^
*k*_2_	0.22 μm/s	(S4B) 0.33; (7A) 0.25; (7B) 0.72
*k*_3a_	0.45 s^−1^	(6A) 0.77; (6B) 0.65; (S4A) 0.42; (S4B) 0.57; (7A) 0.7; (7B) 0.71; (8B) 0.39
*k*_11_	4∙10^−3^ μm/s	(S4B) 6∙10^−3^; (7A) 4.6∙10^−3^; (7B) 6.6∙10^−3^; (8B) 3∙10^−3^
*k*_12_	6∙10^−3^ μm/s	(7A) 5.6∙10^−3^; (7B) 7∙9^−3^; (8B) 5∙10^−3^
*k*_5_	0.095 μm/s	(7B) 0.126
*k*_6a_	0.6 s^−1^	(7B) 0.393
*k*_6b_	0.43 s^−1^	(7B) 0.157
*k*_4_	0.24 s^−1^	(7B) 0.11
*k*_41_	0.8∙10^−3^ μm/s	(7B) 0.22; (8B) 0.4∙10^−3^
*k*_42_	3.6∙10^−3^ μm/s	(7B) 1∙10^−3^; (8B) 2∙10^−3^
*L*	40 μm	(S4A) 60; (S4B) 60
*N*_Rac1_	5200	(4A) 4500; (4B) 5000; (6A) 7000; (6B) 6000; (6C) 6200; (S4A) 8580; (S4B) 8100; (S4C) 6000; (7A) 7300; (7B) 6340; (8B) 5400
*N*_GAP_	1200	(6A) 1000; (S4A) 2040; (S4B) 1380; (S4C) 1100; (7A) 1600; (7B) 1360; (8B) 1440
*N*_DGAP1_	1600	(4A) 800; (4B) 800; (6A) 900; (6B) 900; (6C) 3400; (S4A) 1320; (S4B) 1440; (S4C) 4400; (7A) 1100; (7B) 1530; (8B) 1460
**Additional parameters in extensive model**		
*k*_−1_	0.85 s^−1^	
*k*_act_	1 s^−1^	
*k*_−4_	0.05 s^−1^	
*k*_6c_	0.3 s^−1^	
*k*_6d_	0.3 s^−1^	
*k*_3b_	0.25 s^−1^	

First column: parameter symbols. Second column: initial estimates of parameter values. Third column: adjusted values corresponding to specific patterns. Each value refers to the corresponding figure label, e.g. (S4B) corresponds to S4B Fig. If a figure label is not quoted in the table, this means that the pattern for that figure was determined using the initial estimate from the second column. Parameters specific to the extensive model, required to account for reverse reactions and alternative dissociation pathways, are listed under ’Additional parameters in extensive model,’ with values provided for each. Also, the following parameter values were used in numerical simulations of the extensive model to obtain the solutions equivalent to the solutions obtained by the reduced model using initial parameter values: $N_{Rac1}=9200,\;N_{GAP}=880,\;N_{DGAP1}=1500$, and *k*_11_ = 5∙10^−3^ μm/s.

### Two-dimensional computational model of Rac1 dynamics

**Fig 9:**
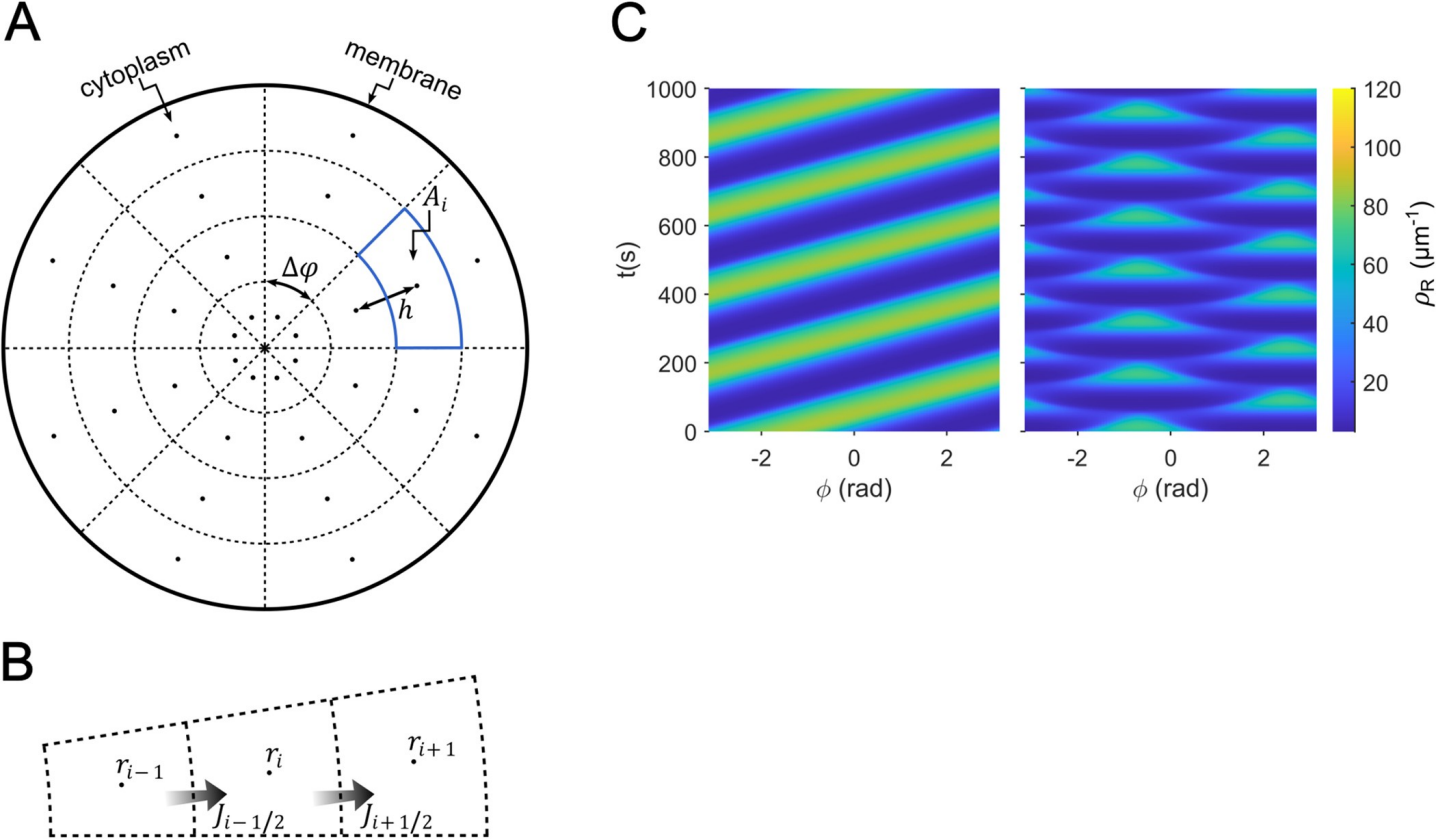
2D simulation of Rac1 dynamics on a disk. (**A**) Disk segmentation. (**B**) Radial diffusion flow between neighbouring segments. (**C**) Results of simulations: L*eft* Travelling waves with *N*_Rac1_ = 7600; *Right* Standing waves with *N*_Rac1_ = 6900. All other parameter values are the same in both simulations: *ρ*_Rmax_ = 200, $k_{1}^{\prime }=1.6∙10^{-2}$, $k_{2}^{\prime }=2.5$, $k_{3}=0.3$, $k_{11}^{\prime }=3.2∙10^{-3}$, $k_{12}^{\prime }=4.8∙10^{-3}$, *R* = 6, *D*_r_ = 30, *D*_g_ = 8, *N*_GAP_ = 2000.

To verify whether the wave-like solutions from our one-dimensional (1D) computational model are also generated by an analogous two-dimensional (2D) model, we performed numerical simulations of the left-hand side of the model (Eqs 1–4) using a finite difference method applied to a 2D disk to model reaction and diffusion processes. The disk is segmented into *n* concentric rings (radial segments) and each ring is further divided into *m* angular segments (Fig 9A). The radial positions inside the disk are given by *r*_*i*_ = (2*i*−1)/*h*/2 for *i* = 1,…,*n*, where *h* denotes the distance between two adjacent radial positions. Angular positions are *φ*_*j*_ = (2*j*−1)Δ*φ*/2 for *j* = 1,…,*m*. The continuous function *ρ*(*r*,*φ*,*t*) representing 2D cytoplasmic concentration is approximated at discrete points (*r*_*i*_,*φ*_*j*_) as *ρ*_*i*,*j*_(*t*), which is also the average concentration within the entire *ij*-th segment. The membrane is represented by the outermost ring at the radial position *nh*. The 1D membrane concentration is approximated at points (*nh*,*φ*_*j*_) as *ρ*_*j*_(*t*).

### Central difference approximation

To model the diffusion of molecules within the cytoplasm, we apply the Laplace operator to the concentration field *ρ* in polar coordinates. In the continuous form, the Laplace operator ∇^2^ applied to *ρ* is given by:

$$\nabla^{2}\rho =\frac{\partial^{2}\rho }{\partial r^{2}}+\frac{1}{r}\frac{\partial \rho }{\partial r}+\frac{1}{r^{2}}\frac{\partial^{2}\rho }{\partial \varphi^{2}}.$$

For the radial part, the approximation of the 2nd derivative is:

$${\frac{\partial^{2}\rho }{\partial r^{2}}|}_{r_{i},\varphi_{j}}\approx \frac{1}{h^{2}}(\rho_{i+1,j}-2\rho_{i,j}+\rho_{i-1,j}),$$

and the approximation of the 1st derivative is:

$${\frac{\partial \rho }{\partial r}|}_{r_{i},\varphi_{j}}\approx \frac{1}{2h}(\rho_{i+1,j}-\rho_{i-1,j}).$$

For the angular part:

$${\frac{\partial^{2}\rho }{\partial \varphi^{2}}|}_{r_{i},\varphi_{j}}=\frac{1}{{\Delta \varphi }^{2}}(\rho_{i,j+1}-2\rho_{i,j}+\rho_{i,j-1}).$$

Combining the above terms, the discretized Laplace operator (*L*_*d*_) can be expressed as:

$${\left(\frac{\partial^{2}\rho }{\partial r^{2}}+\frac{1}{r}\frac{\partial \rho }{\partial r}+\frac{\partial^{2}\rho }{\partial \varphi^{2}}\right)|}_{r_{i},\varphi_{j}}\approx \frac{1}{h^{2}}(\rho_{i+1,j}-2\rho_{i,j}+\rho_{i-1,j})+\frac{1}{2r_{i}h}(\rho_{i+1,j}-\rho_{i-1,j})+\frac{1}{r_{i}^{2}\Delta \varphi^{2}}(\rho_{i,j+1}-2\rho_{i,j}+\rho_{i,j-1})=\frac{1}{r_{i}h^{2}}\left[r_{i+1/2}(\rho_{i+1,j}-\rho_{i,j})-r_{i-1/2}(\rho_{i,j}-\rho_{i-1,j})\right]+\frac{1}{r_{i}^{2}\Delta \varphi^{2}}(\rho_{i,j+1}-2\rho_{i,j}+\rho_{i,j-1})=L_{d}\rho_{i,j},$$

where *r*_*i*−1/2_ = *r*_*i*_−*h*/2 represents the position of the radial segment’s inner boundary, and *r*_*i*+1/2_ = *r*_*i*_+*h*/2 represents the position of the radial segment’s outer boundary. Therefore, the change of the concentration *ρ*_*i*,*j*_ due to diffusion is:

$$\frac{\partial \rho_{i,j}}{\partial t}=DL_{d}\rho_{i,j}.$$

### Formulation in terms of radial fluxes

Radial diffusion fluxes through the *ij*-th segment are (Fig 9B):

$$J_{i-1/2,j}=-\frac{D}{h}(\rho_{i,j}-\rho_{i-1,j}),$$

$$J_{i+1/2,j}=-\frac{D}{h}(\rho_{i+1,j}-\rho_{i,j}),$$

where *J*_*i*−1/2,*j*_ is the flux through the segment’s inner boundary and *J*_*i*+1/2,*j*_ is the flux through the segment’s outer boundary.

The radial part of the diffusion equation can now be written in terms of radial fluxes:

$$\frac{D}{r_{i}h^{2}}\left[r_{i+1/2}(\rho_{i+1,j}-\rho_{i,j})-r_{i-1/2}(\rho_{i,j}-\rho_{i-1,j})\right]=\frac{1}{r_{i}h}(r_{i-1/2}J_{i-1/2,j}-r_{i+1/2}J_{i+1/2,j})=\frac{\Delta \varphi }{A_{i}}(r_{i-1/2}J_{i-1/2}-r_{i+1/2}J_{i+1/2})=\frac{1}{A_{i}}({\Delta l}_{i-1/2}J_{i-1/2,j}-{\Delta l}_{i+1/2}J_{i+1/2,j}),$$

where *A*_*i*_ = *r*_*i*_*h*Δ*φ* denotes the area of the *ij*-th segment, Δ*l*_*i*−1/2_ = *r*_*i*−1/2_Δ*φ* is the length of the segment’s inner boundary, and Δ*l*_*i*+1/2_ = *r*_*i*+1/2_Δ*φ* is the length of the segment’s outer boundary.

Consequently, beginning with the discrete Laplace operator, we arrive at the expected result that the change in concentration in a particular segment due to diffusion in the radial direction is equal to the total radial flow of molecules through that segment divided by its area. This flux-based approach generalizes the Laplace operator and simplifies modeling the behavior at the cytoplasm-membrane interface, where the outermost cytoplasmic segment’s flux *J*_*n*+1/2_ arises from interactions of molecules with the membrane rather than diffusion. For example, the interaction of cytoplasmic Rac1 molecules with the membrane generates flux in the *nj*-th segment equal to:

$$J_{n+1/2,j}^{Rac1}={\left(\rho_{r}\right)}_{n,j}\left[1-\frac{{\left(\rho_{R}\right)}_{j}}{\rho_{Rmax}}\right][k_{1}^{\prime }+k_{11}^{\prime }{\left(\rho_{R}\right)}_{j}+k_{12}^{\prime }{\left(\rho_{RG}\right)}_{j}]-k_{3a}{\left(\rho_{RG}\right)}_{j}-k_{6b}{\left(\rho_{RD}\right)}_{j}.$$

In this case, instead of the 1D concentration (*ρ*_r_)_*j*_ from the 1D model, we consider the 2D concentration (*ρ*_r_)_*n*,*j*_, which necessitates different rate constants denoted with primed symbols. To establish the relationship between the constants in the 2D and 1D models, we can equate the corresponding fluxes:

$${\left(\rho_{r}\right)}_{n,j}\left[1-\frac{{\left(\rho_{R}\right)}_{j}}{\rho_{Rmax}}\right][k_{1}^{\prime }+k_{11}^{\prime }{\left(\rho_{R}\right)}_{j}+k_{12}^{\prime }{\left(\rho_{RG}\right)}_{j}]-k_{3a}{\left(\rho_{RG}\right)}_{j}-k_{6b}{\left(\rho_{RD}\right)}_{j}={\left(\rho_{r}\right)}_{j}\left[1-\frac{{\left(\rho_{R}\right)}_{j}}{\rho_{Rmax}}\right][k_{1}+k_{11}{\left(\rho_{R}\right)}_{j}+k_{12}{\left(\rho_{RG}\right)}_{j}]-k_{3a}{\left(\rho_{RG}\right)}_{j}-k_{6b}{\left(\rho_{RD}\right)}_{j}.$$

This relationship implies that the primed constants are related to the original constants as follows:

$$k^{\prime }=k\frac{{\left(\rho_{r}\right)}_{j}}{{\left(\rho_{r}\right)}_{n,j}}.$$

### Complete finite-difference scheme

For *i* = 1,…,*n*−1:

$$\frac{\partial \rho_{i,j}}{\partial t}=\frac{D}{r_{i}h^{2}}\left[r_{i+1/2}(\rho_{i+1,j}-\rho_{i,j})-r_{i-1/2}(\rho_{i,j}-\rho_{i-1,j})\right]+D\frac{1}{r_{i}^{2}\Delta \varphi^{2}}(\rho_{i,j+1}-2\rho_{i,j}+\rho_{i,j-1}).$$

For *i* = *n*:

$$\frac{\partial \rho_{n,j}}{\partial t}=-\frac{D}{r_{n}h^{2}}r_{n-1/2}(\rho_{n,j}-\rho_{n-1,j})-\frac{1}{r_{n}h}r_{n+1/2}J_{n+1/2,j}+D\frac{1}{r_{n}^{2}\Delta \varphi^{2}}(\rho_{n,j+1}-2\rho_{n,j}+\rho_{n,j-1}).$$

At the membrane (without membrane diffusion):

$$\frac{\partial \rho_{j}}{\partial t}=J_{n+1/2,j}.$$

### Simulation results

For simplicity, we simulated only the part of our model that includes the interaction between Rac1 and GAP, and obtained qualitatively similar results for the Rac1 dynamics to those from the 1D version of the model, including traveling and standing wave solutions (Fig 9C). This result supports the 1D model’s ability to capture the dynamics of the simulated system while significantly reducing computational complexity.

### Sensitivity analysis

The influence of model parameters on the *ρ*_R_ traveling wave speed, *v*, was assessed by conducting a local sensitivity analysis using the normalized sensitivity coefficient *S*. This coefficient measures the relative change in wave speed with respect to the relative change in the parameter value:

$$S_{v}=(\Delta v/v_{0})/(\Delta p/p_{0}),$$

where Δ*v* = *v*−*v*_0_ represent the change in wave speed, *v*_0_ is the reference wave speed (at the baseline parameter value), and Δ*p* = *p*−*p*_0_ denotes the change in the parameter value relative to the baseline parameter value *p*_0_. *Note*: Varying *ρ*_Rmax_, which represents the maximum concentration of binding sites for the species Rac1_T_, needs to be accompanied by corresponding changes in the parameters *k*_1_,*k*_11_, and *k*_12_. This is because these rate constants are scaled with *ρ*_Rmax_. For example, *k*_1_ is defined as $k_{1}=k_{1}^{\prime }\rho_{Rmax}$, where $k_{1}^{\prime }$ represents the intrinsic rate constant, which is independent of the binding site concentration. By scaling the rate constants with *ρ*_Rmax_, we ensure that the model accurately captures the relationship between the binding site availability and the overall reaction rates. Similar holds for *ρ*_Dmax_.

The wave speed was determined using linear stability analysis. The perturbation ansatz $\delta \rho (x,t)\propto e^{\sigma_{q}t+iqx}$ was equated with the travelling wave ansatz *δρ*(*z*)∝*e*^*iqz*^, where *z* = *x*−*vt* represents spatial coordinate in a moving reference frame (moving along with the wave). Thus:

$$e^{\sigma t+iqx}=e^{iqz}=e^{iq(x-vt)},$$

from which follows:

σ=−iqv.

Considering that *σ*_*q*_ is a complex number, it can be written as:

Reσ+iImσ=−iqv.

To determine the wave speed, it is necessary to find a wave number *q*_*m*_ for which the real part of the growth rate, Re*σ*(*q*_*m*_), equals zero. This specific wave number is referred to as the marginally stable wave number. By setting Re*σ*(*q*_*m*_) = 0, we derive the expression for the wave speed: *v* = Im*σ*(*q*_*m*_)/*q*_*m*_. Consequently, linear stability analysis predicts that the speed of the traveling wave is governed by the marginally stable mode.

### Principal component analysis

To compare complicated phase relationships between the observed waves of active Rac1 and DGAP1, we simplified the complexity of the raw fluorescence intensity data using principal component analysis (PCA). This allowed us to determine the average temporal evolution of the fluorescence signals for DPAKa(GBD)-DYFP and mRFP-DGAP1, which we then used to generate phase portraits. For PCA, we designated *I*_Rac1*_ and $I_{{DGAP1}^{\#}}$ as the fluorescence intensity matrices for Rac1* and DGAP1^#^ probes, respectively. These two *n*×*m* matrices, where *n* is the number of time points and *m* is the number of spatial points sampled along the cell membrane, were concatenated vertically to form a 2*n*×*m* matrix *X*. In PCA, we consider each column vector within the matrix *X* as a data point in a 2*n*-dimensional space. We then standardize the data, i.e. we calculate the distance of each data point from the mean in terms of the standard deviation. The rows of the standardized data matrix *Z* are given by:

$$Z_{i,*}=\frac{X_{i,*}-\mu (X_{i,*})}{\sigma (X_{i,*})}$$

where the subscript *i*,* denotes the *i*^th^ row of a matrix; *μ* stands for the mean value and *σ* for the standard deviation.

**Fig 10:**
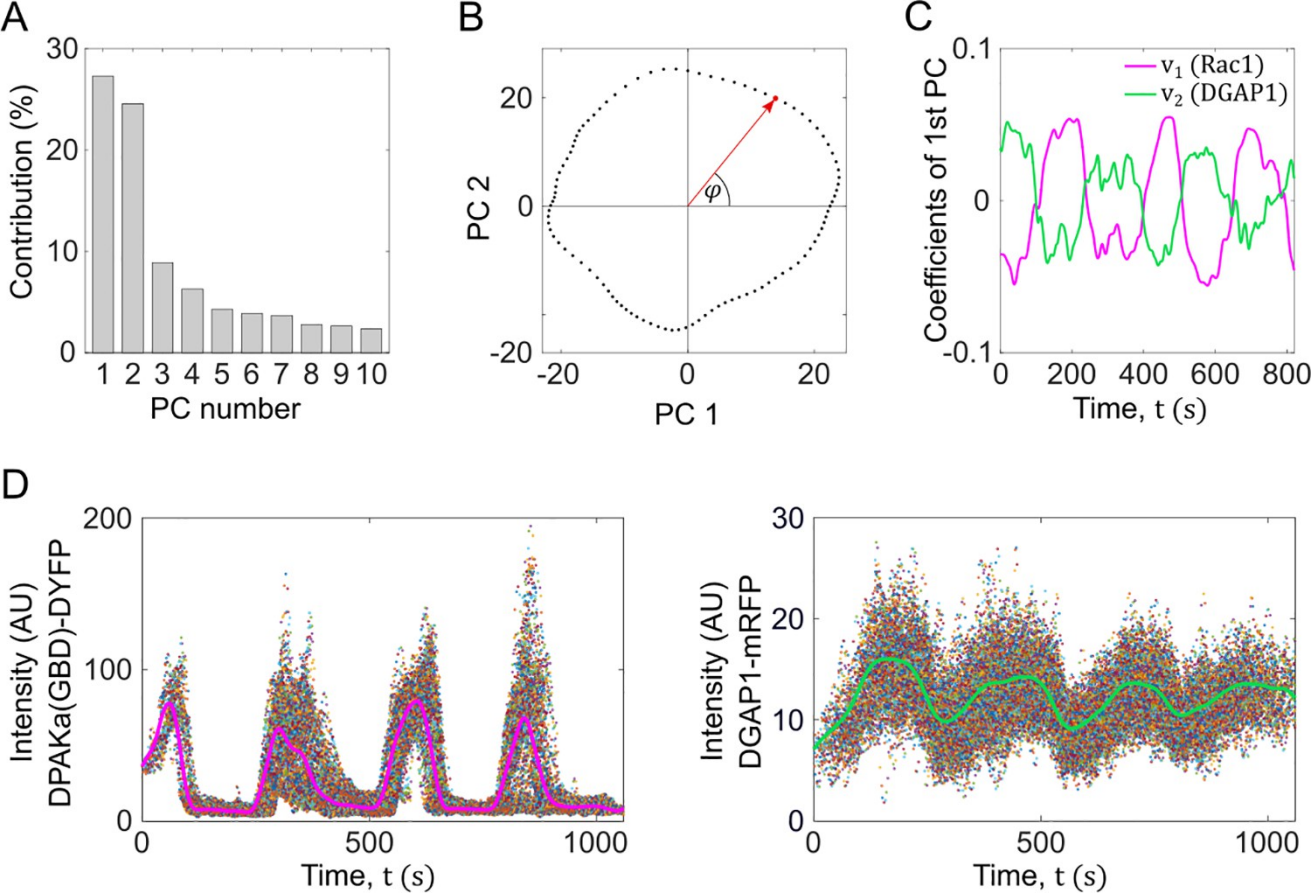
Example of the application of principal component analysis to a traveling wave pattern. (**A**) The contributions of the first 10 principal components to the total variance of the data set are given in percent. The first two components account for more than 50% of the variance; therefore, we use them to characterize the dynamics of DPAKa(GBD)-DYFP and mRFP-DGAP1. (**B**) The projection of the matrix Z onto the first two principal components, denoted as (PC1, PC2), shows the phase relationships between all data points. By shifting each point *c*_*j*_ by an angle of −*φ*_*j*_, we obtain an aligned data set. (**C**) The coefficients of the first principal component are shown as time series for DPAKa(GBD)-DYFP (*v*_1_) and mRFP-DGAP1 (*v*_2_). (**D**) The aligned time series of fluorescence intensities for DPAKa(GBD)-DYFP (*left*) and mRFP-DGAP1 (*right*). Each color corresponds to a time series associated with a specific point on the membrane. The thick lines represent the average temporal evolution of the signal.

Next, we determine the principal components of the standardized data matrix *Z* using the MATLAB function ’pca’. The input of the function ’pca’ is the matrix *Z*, and the output is a 2*n*×2*n* matrix *C*, where each column contains the coefficients for one principal component. These columns are sorted in descending order according to the variance of the components as shown in a representative example of the percentage of variation accounted for by the first 10 principal components (Fig 10A). It can be clearly seen that the majority of the information about the dynamics of the Rac1* and DGAP1^#^ probes is contained in the first two principal component axes. We, therefore, reduce the dimensionality of the data by projecting each data point onto a plane defined by the first two principal component axes. The calculations of these projections are performed as follows:

$$c_{1,j}=Z_{*,j}∙C_{*,1}\;and\;c_{2,j}=Z_{*,j}∙C_{*,2},$$

where *c*_1,*j*_ and *c*_2,*j*_ are the projections of the *j*^th^ data point onto the first and second principal component axes, respectively. By examining the projected data, it is clear that PCA not only preserves the periodicity of the Rac1_T_ and DGAP1 dynamics, but also elucidates the phase relationships for all points in the data set (Fig 10B). We can use this information to shift all data points to create a new aligned dataset. From this dataset, we can derive the average temporal evolution of the system. The time shift for the *j*^th^ point is given as follows:

$$\Delta t_{j}=-T\varphi_{j}/2\pi ,$$

where *φ*_*j*_ represents the phase angle and *T* symbolizes the period. To estimate *T*, we use the coefficients of the first principal component. We interpret the vector *v*_1_ = (*C*_1,1_,…,*C*_*n*,1_) as a time series of the DPAKa(GBD)-DYFP fluorescence intensity values and the vector *v*_2_ = (*C*_*n*+1,1_,…,*C*_2*n*,1_) as a time series of the mRFP-DGAP1 fluorescence intensity values (Fig 10C). Both *v*_1_ and *v*_2_ obviously show oscillatory behavior, so we use them to calculate the period *T*. The aligned data sets with thick lines indicating the average dynamics are shown in Fig 10D.

### Noise analysis of the Rac1* signal

To analyze the noise characteristics and the spatial dynamics of the Rac1 activity, we used the intensity matrix *I*_Rac1*_. Unlike in other sections where smoothing was applied, here no smoothing was performed on the data. Instead, the original Rac1* intensity data, which were sampled at irregularly spaced points on the membrane, were interpolated onto a regular grid of spatial points to standardize the spatial resolution across time. The analysis of noise included three steps:

#### 1) Preprocessing and variance-stabilizing transformation

The intensity matrix *I*_Rac1*_ was first preprocessed to account for potential intensity-dependent noise. Specifically, the square root of the raw intensity values was taken:

$$I_{{Rac1}^{*}}^{sqrt}=\sqrt{I_{{Rac1}^{*}}}.$$

This transformation reduces the heteroscedasticity commonly present in fluorescence data, particularly in regions with high fluorescence intensity.

#### 2) High-pass filtering

To isolate the noise from low-frequency spatial patterns, we applied a high-pass Butterworth filter to the transformed intensity matrix. The filter was designed using MATLAB’s ’butter’ function, which returns the filter coefficients *b* (numerator) and *a* (denominator). A 4^th^ order high-pass Butterworth filter was used, with a cutoff frequency *f*_*c*_ = 0.15 cycles per unit length. This cutoff frequency was chosen to remove low-frequency patterns while preserving high-frequency noise. The filter was applied using MATLAB’s ’filtfilt’ function, which performs zero-phase filtering by running the filter forward and backward across the data to avoid phase distortion. The filtering was applied to each time point in the matrix using the following equation:

$$I_{{Rac1}^{*}}^{filtered}(t,x)=filtfilt(b,a,I_{{Rac1}^{*}}^{sqrt}),$$

where *t* represents time and *x* represents the spatial position across the cell membrane.

#### 3) Power spectral density calculation

The filtered intensity matrix $I_{{Rac1}^{*}}^{filtered}$ was then subjected to spectral analysis using the Fast Fourier Transform (FFT) to compute the Power Spectral Density (PSD). For each time point, the spatial frequency content of the Rac1 activity was calculated, and the single-sided PSD was obtained by averaging across all time points. The PSD allowed for the characterization of noise in the system across different spatial frequencies:

$${PSD}_{{Rac1}^{*}}(f)=\frac{2}{N}{\left|FFT(I_{{Rac1}^{*}}^{filtered})\right|}^{2},$$

where *f* is the spatial frequency and *N* is the number of spatial points.

## Supporting information

S1 Fig Representative bipolar patterns of cortical domains enriched in Rac1*. (**A**) *Rotating dipole*. Rotation of two different domains. (**B**) *Oscillating dipole*. Two domains located on opposite sides and periodically changing their orientation. (**C**) *Stationary dipole*. Two domains located on opposite sides remain stationary for several minutes. Scale bars: 5 μm.
(PDF)

S2 Fig A representative oscillating dipole in a double-labeled cell. The sequence shown encompasses a complete oscillatory cycle. Top: two domains enriched in Rac1*. Bottom: two complementary domains enriched in DGAP1^#^. Scale bar: 5 μm.
(PDF)

S3 Fig Impact of diffusion coefficients on pattern formation in the Rac1-GAP system. Stability diagram shows different dynamic regimes as a function of diffusion coefficients of Rac1_D_ (*D*_r_) and GAP (*D*_g_). Turing region is shown in blue, Hopf region in red, Turing-Hopf region in green, and stable homogeneous states in white. All other parameters were held constant and their values are listed in Table 2.
(PDF)

S4 Fig Comparison of the experimentally observed patterns of Rac1* with the patterns of Rac1_T_ derived from computer simulations. In panel A, the patterns are shown as kymographs (top) and the corresponding autocorrelograms (bottom). In panels B-C only the kymographs are shown. Kymographs and autocorrelograms are defined as described in Fig 4. The patterns shown correspond to the dynamics types shown in S1 Fig: (**A**) *Oscillating dipole*, (**B**) *Rotating dipole*, and (**C**) *Stationary dipole*. The parameter values used in the simulations are listed in Table 2.
(PDF)

S5 FigEffect of circumference size on pattern formation.(**A**) The growth rate has positive values for wavenumbers within the interval (0,*q*_*m*_), implying that these modes are unstable. Since the wavenumber is a function of *L*, represented as *q* = 2*nπ*/*L*, the value *q*_*m*_ defines the minimum length, $L_{n}^{min}=2n\pi /q_{m}$, at which the *n*^th^ Fourier mode becomes unstable. Using the parameter values in Table 2, we have determined $L_{1}^{min}∼20\;\mu m,\;L_{2}^{min}∼40\;\mu m$, and so on. As *L* increases (which means *q* decreases), the type of instability changes from oscillatory to stationary, as indicated by the kink in the growth curve. For all $L>L_{1}^{min}$, however, the result of the simulations is an oscillating pattern. (**B**) A comparison of the measured perimeters of cells showing first-order oscillatory patterns (Osc m1), second-order oscillatory patterns (Osc m2), and first-order stationary patterns (Pol m1). The red lines denote the respective median values: *L*(Osc_m1) = 38 (36 to 41) μm, *n* = 31; *L*(Osc_m2) = 48 (42 to 51) μm, *n* = 15; *L*(Pol_m1) = 43 (40 to 45) μm, *n* = 9, (median, interquartile range); * means *P*<0.01, while n.s. means not significant.(PDF)

S6 Fig Representative power spectral density of the Rac1 activity. The plot shows the average power spectral density (PSD) of the filtered Rac1* intensity matrix (*I*_Rac1*_​) across spatial frequencies for one typical cell, chosen to represent the general behavior observed across multiple cells. The y-axis represents the power per unit frequency, in the units of (a.u.)^2^/cycles per micrometer. The x-axis denotes the spatial frequency in cycles per micrometer. The concentration of power at low spatial frequencies suggests that noise in the system is dominated by large-scale spatial fluctuations. Higher spatial frequencies show a rapid decay in power, indicating reduced noise at smaller spatial scales. For the details of calculation, see Materials and methods section Noise analysis of the Rac1* signal.
(PDF)

S7 Fig Monopolar patterns of Rac1* in DGAP1/GAPA knock-out cells. (**A**) *Rotating monopole* Selected frames show the rotation of a membrane domain enriched in Rac1*. (**B**) *Oscillating monopole* Selected frames show the periodic movement of a Rac1* domain from one to the opposite side of the cell. These observations show that the absence of DGAP1 and the closely related protein GAPA does not prevent the formation of rotating and oscillating patterns of Rac1 activity. Scale bars: 5 μm.
(PDF)

S1 Movie Representative rotating monopole. A Rac1*-enriched domain travels along the cell membrane (corresponds to Fig 1A).
(AVI)

S2 Movie Representative rotating dipole. Two distinct Rac1*-enriched domains travel along the cell membrane (corresponds to S1A Fig).
(AVI)

S3 Movie Representative oscillating monopole. A Rac1*-enriched domain periodically relocates from one side of the cell to the opposite side (corresponds to Fig 1B).
(AVI)

S4 Movie Representative oscillating dipole. Two distinct Rac1*-enriched domains periodically undergo 90-degree orientation shifts (corresponds to S1B Fig).
(AVI)

S5 Movie Representative stationary monopole. A Rac1*-enriched domain remains on one side of a migrating cell (corresponds to Fig 1C).
(AVI)

S6 Movie Representative stationary dipole. Two distinct Rac1*-enriched domains located on opposite sides of the cell maintain their positions (corresponds to S1C Fig).
(AVI)

S7 Movie Representative rotating monopoles. Rac1* and DGAP1^#^-enriched domains, located on opposite sides of the cell, travel along the cell membrane (corresponds to Fig 2A).
(AVI)

S8 Movie Representative oscillating dipoles. Two distinct Rac1*-enriched domains and oppositely localized DGAP1^#^-enriched domains periodically shift their orientation by 90 degrees (corresponds to S2 Fig).
(AVI)

S9 Movie Representative stationary monopoles out of phase. Rac1*-enriched domain and oppositely localized DGAP1^#^-enriched domain maintain their positions (corresponds to Fig 2B).
(AVI)

S10 Movie Representative stationary monopoles in phase. Rac1*-enriched and DGAP1^#^-enriched domains are co-localized, maintaining their position on the cell membrane (corresponds to Fig 2C).
(AVI)
