# Supplementary material for: Oscillatory dynamics of Rac1 activity in Dictyostelium discoideum amoebae
Source: PLoS Comput Biol. 2024 Dec 9;20(12):e1012025. doi: 10.1371/journal.pcbi.1012025 (PMC11658709; doi:10.1371/journal.pcbi.1012025)
Supplement: S1 Fig — (A) Rotating dipole. Rotation of two different domains. (B) Oscillating dipole. Two domains located on opposite sides and periodically changing their orientation. (C) Stationary dipole. Two domains located on opposite sides remain stationary for several minutes. Scale bars: 5 μm. (PDF) [file pcbi.1012025.s001.pdf]

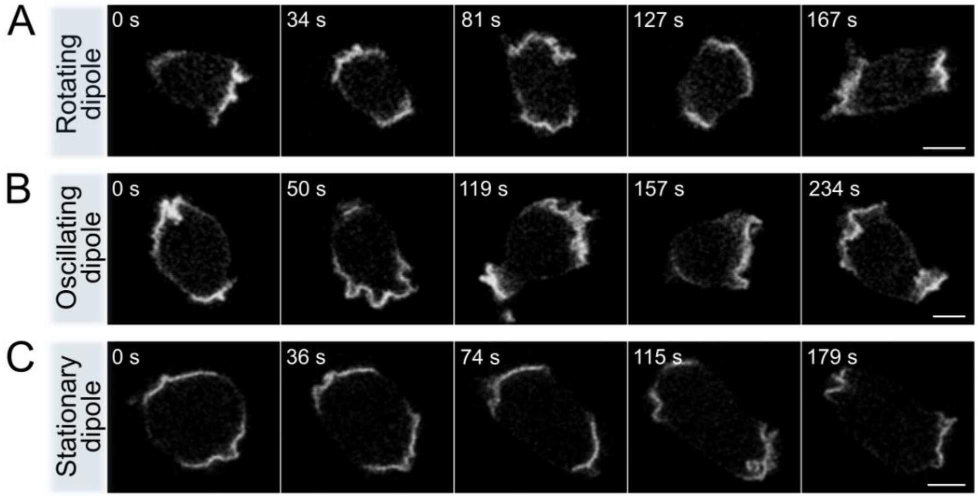

S1 Fig. Representative bipolar patterns of cortical domains enriched in Rac1*. (**A**) *Rotating dipole.* Rotation of two different domains. (**B**) *Oscillating dipole.* Two domains located on opposite sides and periodically changing their orientation. (**C**) *Stationary dipole.* Two domains located on opposite sides remain stationary for several minutes. Scale bars: 5 μm.
