# Supplementary material for: Oscillatory dynamics of Rac1 activity in Dictyostelium discoideum amoebae
Source: PLoS Comput Biol. 2024 Dec 9;20(12):e1012025. doi: 10.1371/journal.pcbi.1012025 (PMC11658709; doi:10.1371/journal.pcbi.1012025)
Supplement: S2 Fig — The sequence shown encompasses a complete oscillatory cycle. Top: two domains enriched in Rac1*. Bottom: two complementary domains enriched in DGAP1#. Scale bar: 5 μm. (PDF) [file pcbi.1012025.s002.pdf]

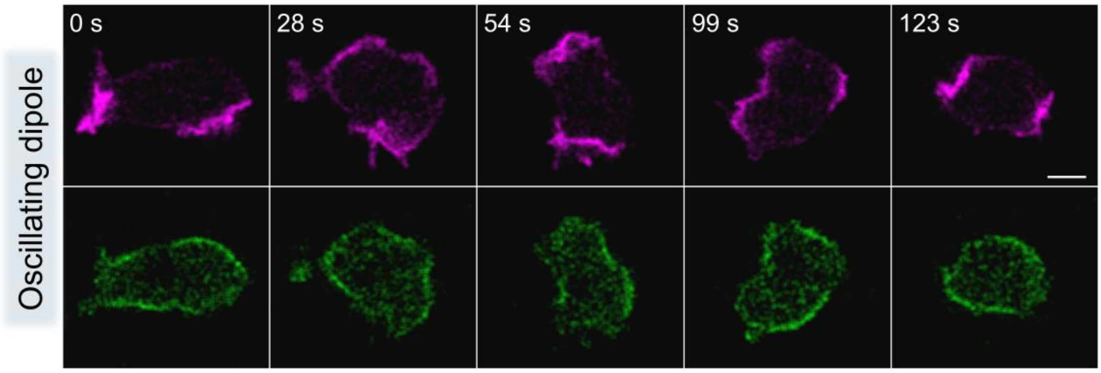

S2 Fig. A representative oscillating dipole in a double-labeled cell. The sequence shown encompasses a complete oscillatory cycle. Top: two domains enriched in Rac1*. Bottom: two complementary domains enriched in DGAP1$^{\#}$. Scale bar: 5 μm.
