# Supplementary material for: Oscillatory dynamics of Rac1 activity in Dictyostelium discoideum amoebae
Source: PLoS Comput Biol. 2024 Dec 9;20(12):e1012025. doi: 10.1371/journal.pcbi.1012025 (PMC11658709; doi:10.1371/journal.pcbi.1012025)
Supplement: S3 Fig — Stability diagram shows different dynamic regimes as a function of diffusion coefficients of Rac1D (Dr) and GAP (Dg). Turing region is shown in blue, Hopf region in red, Turing-Hopf region in green, and stable homogeneous states in white. All other parameters were held constant and their values are listed in Table 2. (PDF) [file pcbi.1012025.s003.pdf]

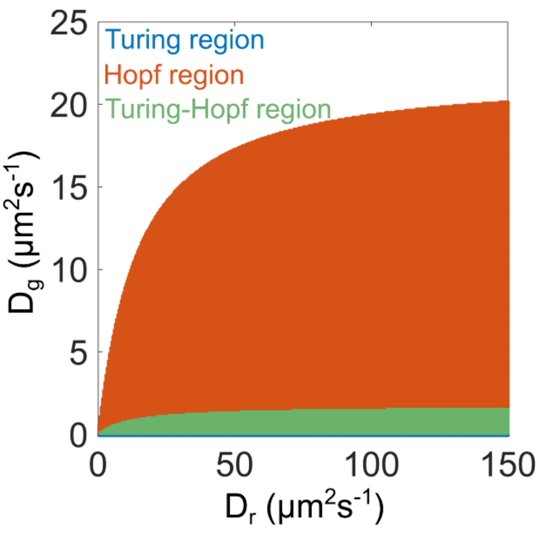

S3 Fig. Impact of diffusion coefficients on pattern formation in the Rac1-GAP system. Stability diagram shows different dynamic regimes as a function of diffusion coefficients of $Rac1_D$ (D_r) and GAP (D_g). Turing region is shown in blue, Hopf region in red, Turing-Hopf region in green, and stable homogeneous states in white. All other parameters were held constant and their values are listed in Table 2.
