# Supplementary material for: Oscillatory dynamics of Rac1 activity in Dictyostelium discoideum amoebae
Source: PLoS Comput Biol. 2024 Dec 9;20(12):e1012025. doi: 10.1371/journal.pcbi.1012025 (PMC11658709; doi:10.1371/journal.pcbi.1012025)
Supplement: S4 Fig — In panel A, the patterns are shown as kymographs (top) and the corresponding autocorrelograms (bottom). In panels B-C only the kymographs are shown. Kymographs and autocorrelograms are defined as described in Fig 4. The patterns shown correspond to the dynamics types shown in S1 Fig: (A) Oscillating dipole, (B) Rotating dipole, and (C) Stationary dipole. The parameter values used in the simulations are listed in Table 2. (PDF) [file pcbi.1012025.s004.pdf]

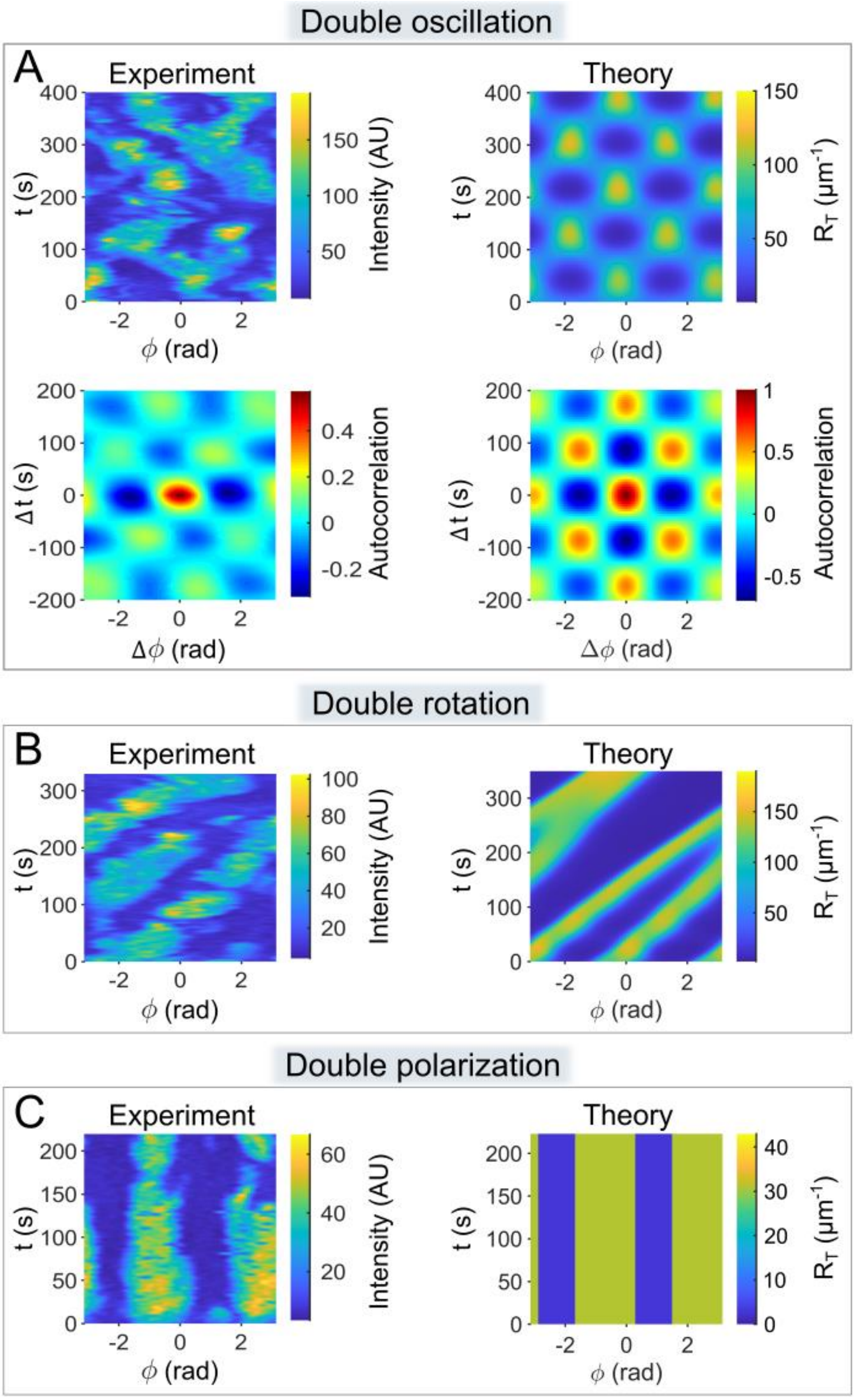

S4 Fig. Comparison of the experimentally observed patterns of Rac1* with the patterns of Rac1$_T$ derived from computer simulations. In panel A, the patterns are shown as kymographs (top) and the corresponding autocorrelograms (bottom). In panels B-C only the kymographs are shown. Kymographs and autocorrelograms are defined as described in Fig 4. The patterns shown correspond to the dynamics types shown in S1 Fig: (**A**) *Oscillating dipole*, (**B**) *Rotating dipole*, and (**C**) *Stationary dipole*. The parameter values used in the simulations are listed in Table 2.
