# Supplementary material for: Oscillatory dynamics of Rac1 activity in Dictyostelium discoideum amoebae
Source: PLoS Comput Biol. 2024 Dec 9;20(12):e1012025. doi: 10.1371/journal.pcbi.1012025 (PMC11658709; doi:10.1371/journal.pcbi.1012025)
Supplement: S5 Fig — (A) The growth rate has positive values for wavenumbers within the interval (0,qm), implying that these modes are unstable. Since the wavenumber is a function of L, represented as q = 2nπ/L, the value qm defines the minimum length, Lnmin=2nπ/qm, at which the nth Fourier mode becomes unstable. Using the parameter values in Table 2, we have determined L1min∼20μm,L2min∼40μm, and so on. As L increases (which means q decreases), the type of instability changes from oscillatory to stationary, as indicated by the kink in the growth curve. For all L>L1min, however, the result of the simulations is an oscillating pattern. (B) A comparison of the measured perimeters of cells showing first-order oscillatory patterns (Osc m1), second-order oscillatory patterns (Osc m2), and first-order stationary patterns (Pol m1). The red lines denote the respective median values: L(Osc_m1) = 38 (36 to 41) μm, n = 31; L(Osc_m2) = 48 (42 to 51) μm, n = 15; L(Pol_m1) = 43 (40 to 45) μm, n = 9, (median, interquartile range); * means P<0.01, while n.s. means not significant. (PDF) [file pcbi.1012025.s005.pdf]

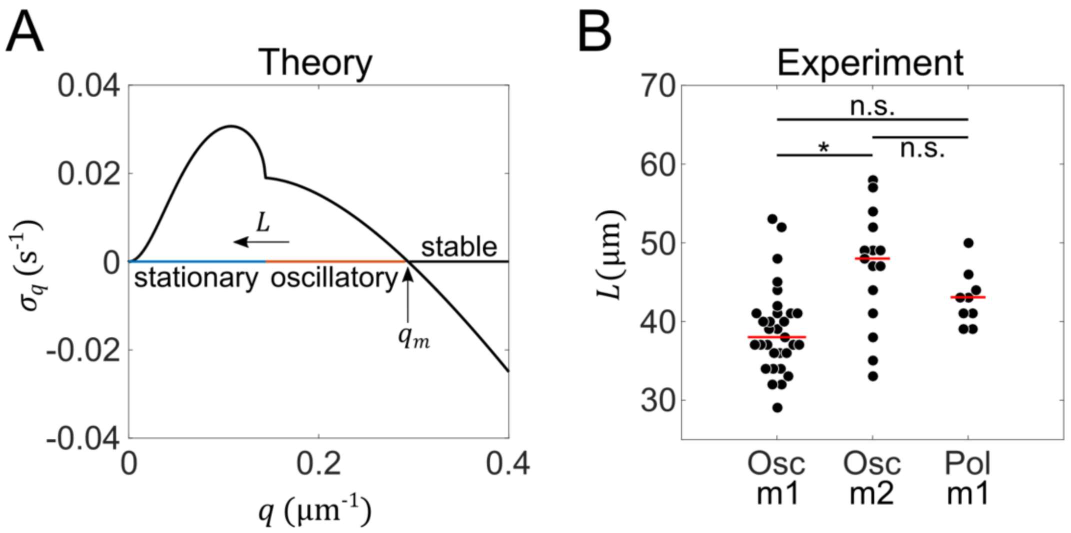

S5 Fig. Effect of circumference size on pattern formation. (**A**) The growth rate has positive values for wavenumbers within the interval $(0, q_m)$, implying that these modes are unstable. Since the wavenumber is a function of L, represented as $q = 2n\pi/L$, the value q_m defines the minimum length, $L_n^{min} = 2n\pi/q_m$, at which the nth Fourier mode becomes unstable. Using the parameter values in Table 2, we have determined L_1^{min}~20 μm, L_2^{min}~40 μm, and so on. As L increases (which means q decreases), the type of instability changes from oscillatory to stationary, as indicated by the kink in the growth curve. For all $L > L_1^{min}$, however, the result of the simulations is an oscillating pattern. (**B**) A comparison of the measured perimeters of cells showing first-order oscillatory patterns (Osc m1), second-order oscillatory patterns (Osc m2), and first-order stationary patterns (Pol m1). The red lines denote the respective median values: $L(\text{Osc\_m1}) = 38$ (36 to 41) μm, $n = 31$; $L(\text{Osc\_m2}) = 48$ (42 to 51) μm, $n = 15$; $L(\text{Pol\_m1}) = 43$ (40 to 45) μm, $n = 9$, (median, interquartile range); * means $P < 0.01$, while n.s. means not significant.
