# Supplementary material for: Oscillatory dynamics of Rac1 activity in Dictyostelium discoideum amoebae
Source: PLoS Comput Biol. 2024 Dec 9;20(12):e1012025. doi: 10.1371/journal.pcbi.1012025 (PMC11658709; doi:10.1371/journal.pcbi.1012025)
Supplement: S6 Fig — The plot shows the average power spectral density (PSD) of the filtered Rac1* intensity matrix (IRac1*​) across spatial frequencies for one typical cell, chosen to represent the general behavior observed across multiple cells. The y-axis represents the power per unit frequency, in the units of (a.u.)2/cycles per micrometer. The x-axis denotes the spatial frequency in cycles per micrometer. The concentration of power at low spatial frequencies suggests that noise in the system is dominated by large-scale spatial fluctuations. Higher spatial frequencies show a rapid decay in power, indicating reduced noise at smaller spatial scales. For the details of calculation, see Materials and methods section Noise analysis of the Rac1* signal. (PDF) [file pcbi.1012025.s006.pdf]

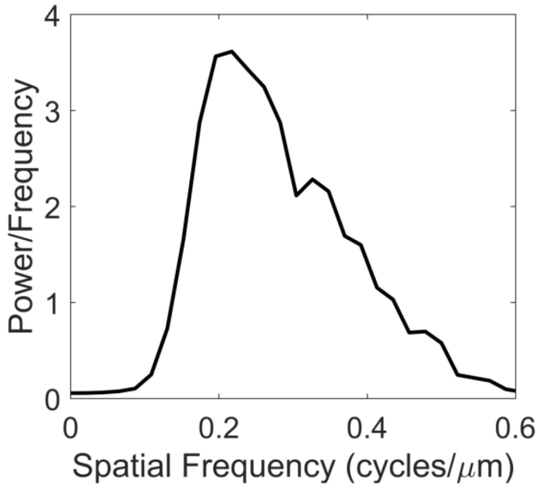

S6 Fig. Representative power spectral density of the Rac1 activity. The plot shows the average power spectral density (PSD) of the filtered Rac1* intensity matrix ($I_{\mathrm{Rac1^*}}$) across spatial frequencies for one typical cell, chosen to represent the general behavior observed across multiple cells. The y-axis represents the power per unit frequency, in the units of $(\mathrm{a.u.})^2/\mathrm{cycles\ per\ micrometer}$. The x-axis denotes the spatial frequency in cycles per micrometer. The concentration of power at low spatial frequencies suggests that noise in the system is dominated by large-scale spatial fluctuations. Higher spatial frequencies show a rapid decay in power, indicating reduced noise at smaller spatial scales. For the details of calculation, see Materials and methods section Noise analysis of the Rac1* signal.
