# Supplementary material for: Oscillatory dynamics of Rac1 activity in Dictyostelium discoideum amoebae
Source: PLoS Comput Biol. 2024 Dec 9;20(12):e1012025. doi: 10.1371/journal.pcbi.1012025 (PMC11658709; doi:10.1371/journal.pcbi.1012025)
Supplement: S7 Fig — (A) Rotating monopole Selected frames show the rotation of a membrane domain enriched in Rac1*. (B) Oscillating monopole Selected frames show the periodic movement of a Rac1* domain from one to the opposite side of the cell. These observations show that the absence of DGAP1 and the closely related protein GAPA does not prevent the formation of rotating and oscillating patterns of Rac1 activity. Scale bars: 5 μm. (PDF) [file pcbi.1012025.s007.pdf]

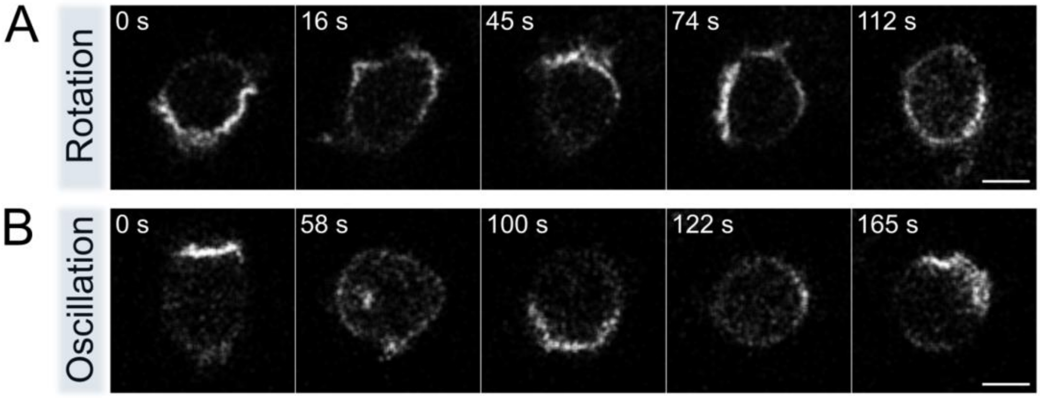

S7 Fig. Monopolar patterns of Rac1* in DGAP1/GAPA knock-out cells. (**A**) *Rotating monopole* Selected frames show the rotation of a membrane domain enriched in Rac1*. (**B**) *Oscillating monopole* Selected frames show the periodic movement of a Rac1* domain from one to the opposite side of the cell. These observations show that the absence of DGAP1 and the closely related protein GAPA does not prevent the formation of rotating and oscillating patterns of Rac1 activity. Scale bars: 5 μm.
